# Supplementary material for: An integrated stress response via PKR suppresses HER2+ cancers and improves trastuzumab therapy
Source: Nat Commun. 2019 May 13;10:2139. doi: 10.1038/s41467-019-10138-8 (PMC6513990; doi:10.1038/s41467-019-10138-8)

An Integrated Stress Response via PKR Suppresses HER2+ cancers and Improves Trastuzumab Therapy.

Darini C,Ghaddar N, Chabot C, Assaker G, Sabri S, Wang S, Krishnamoorthy J, Buchanan M, Aguilar-Mahecha A, Abdulkarim B, Deschenes J, Torres J, Ursini-Siegel J, Mark Basik M, Koromilas AE

SUPPLEMENTARY INFORMATION

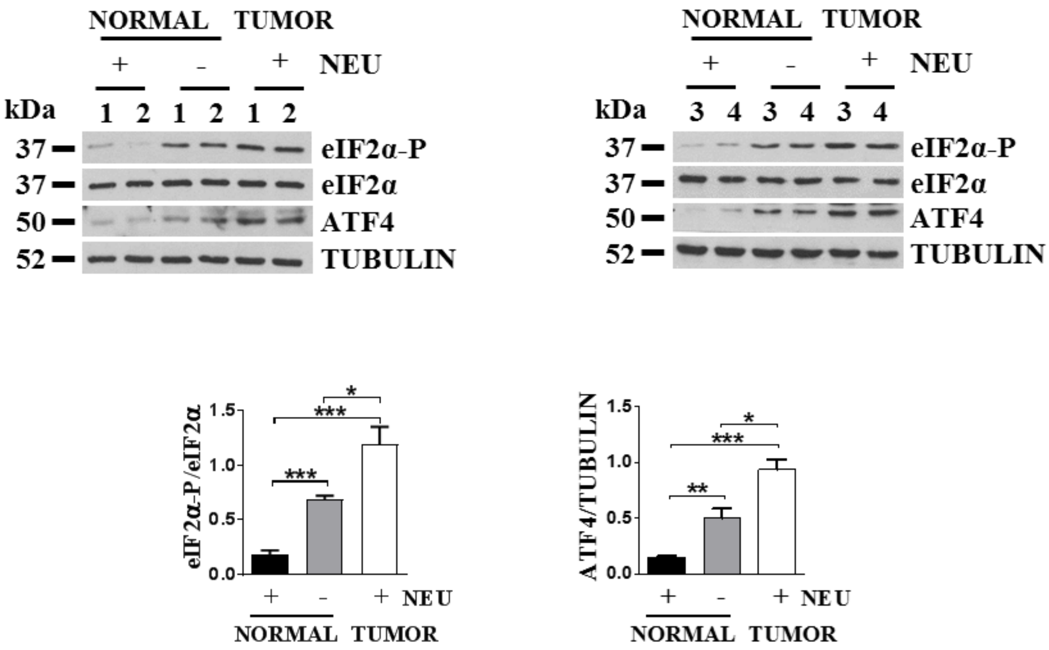

Supplementary Figure 1. eIF2α-P and ATF4 levels are low in mouse NEU mammary gland prior to tumor formation and high in formed tumors. Immunoblotting of protein extracts of mammary tissue from FVB/N mice lacking (Normal, -NEU), or expressing NEU prior to (Normal, +NEU) or after tumor formation (Tumor, +NEU). Quantification of data from the analysis of 4 mice of each group. ±SEM *p<0.05;** p<0.01; *** p<0.001.

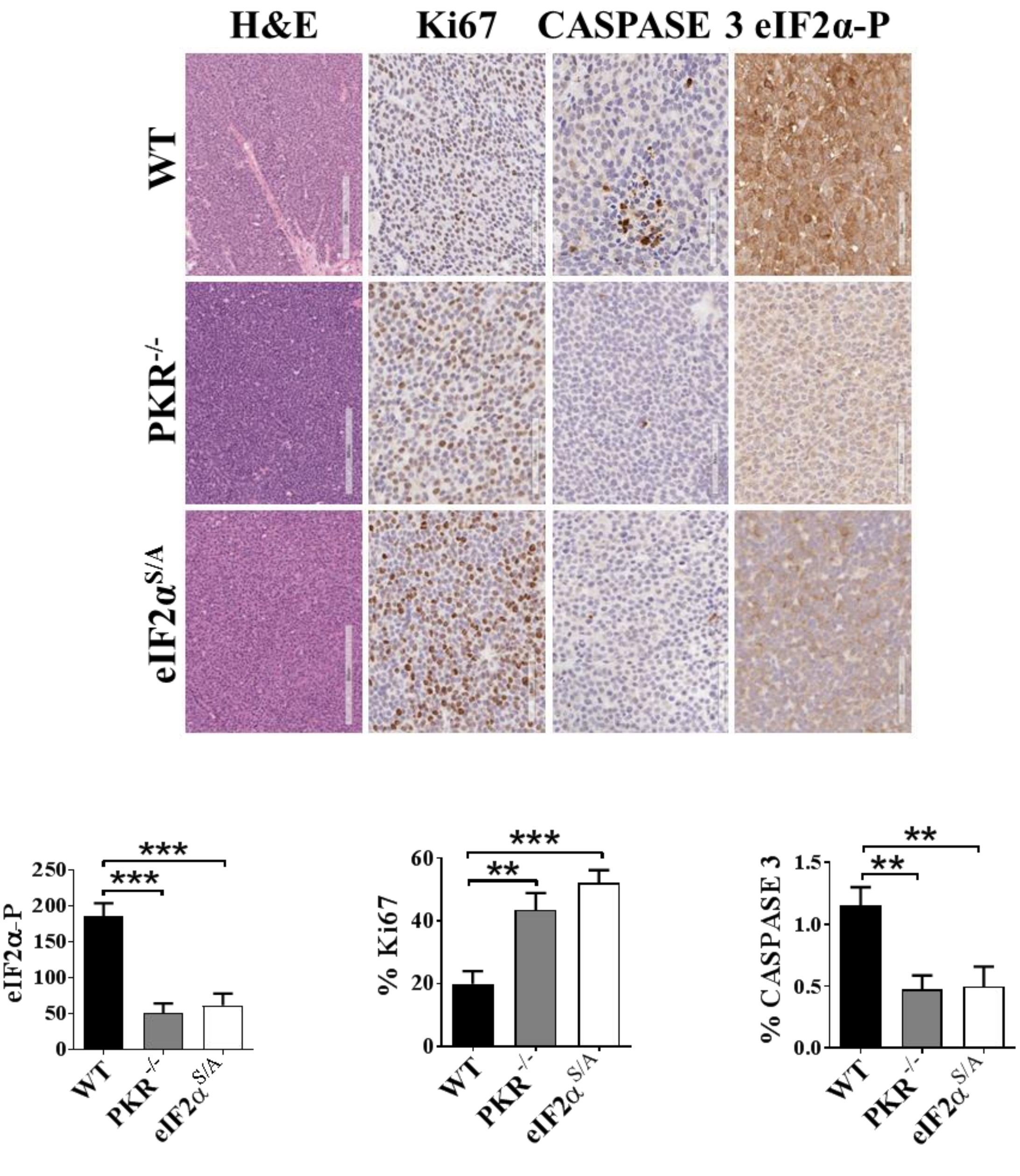

Supplementary Figure 2. PKR and eIF2α-P decrease the proliferation and increase apoptosis of mouse NEU breast tumors. H&E staining and IHC analyses of Ki67, cleaved Caspase 3 and eIF2α-P in sections from NEU WT, NEU $PKR^{-/-}$ or NEU $eIF2\alpha^{S/A}$ tumors isolated 4 weeks after initial detection by palpation (Fig. 1a). The H-score of elF2α-P and the % of positive cells for Ki67 and Cleaved Caspase 3 were quantified by the Aperio ImageScope software from the analysis of tumors from 8 different mice per group. Data represent mean ±SEM ** p<0.01; *** p<0.001.

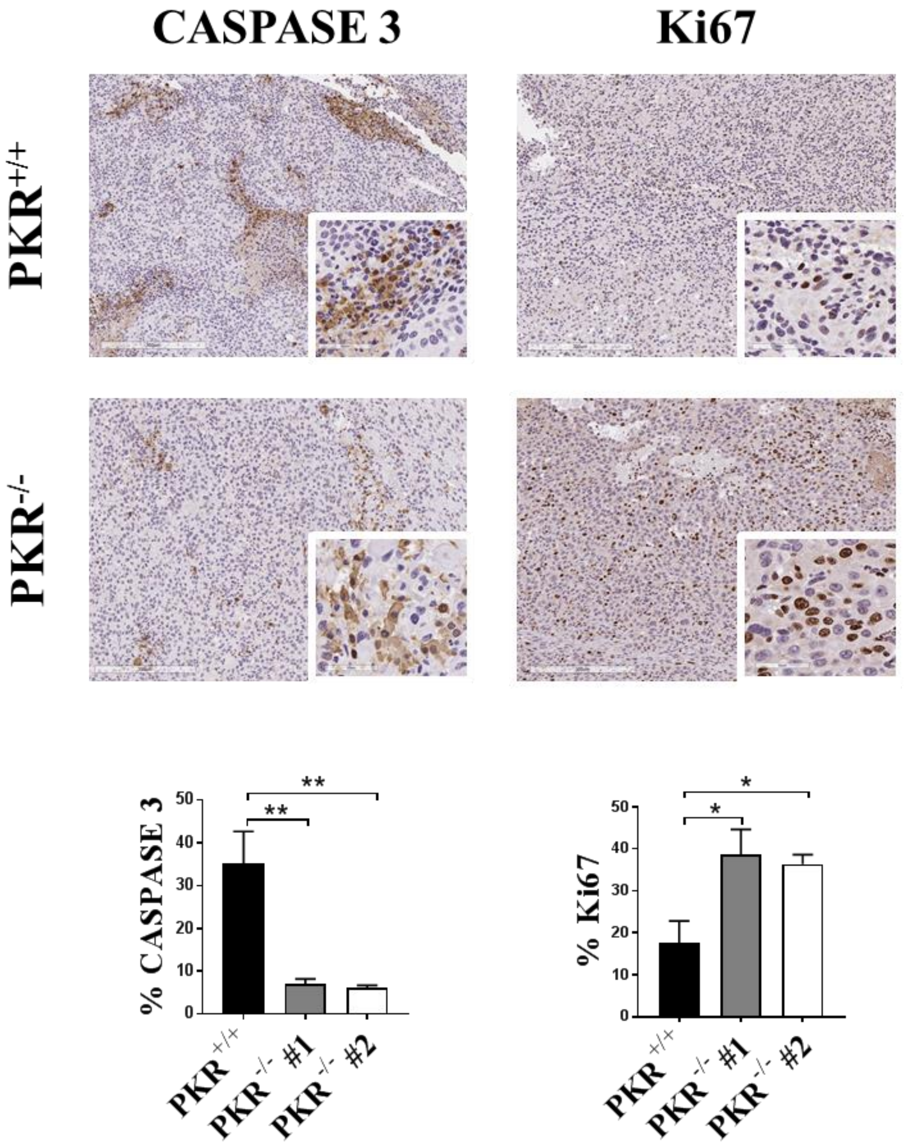

Supplementary Figure 3. PKR inhibits proliferation and increases apoptosis of mouse NEU breast tumors in SCID mice. IHC analyses of Ki67 and cleaved Caspase 3 in sections from NEU WT and NEU PKR$^{-/-}$ tumors isolated from SCID mice (Fig. 2a). The % of positive cells for Ki67 and Cleaved Caspase 3 were quantified by the Aperio ImageScope software from the analysis of tumors from 3 different mice per group. Data represent mean ±SEM $*p < 0.05$; $** p<0.01$.

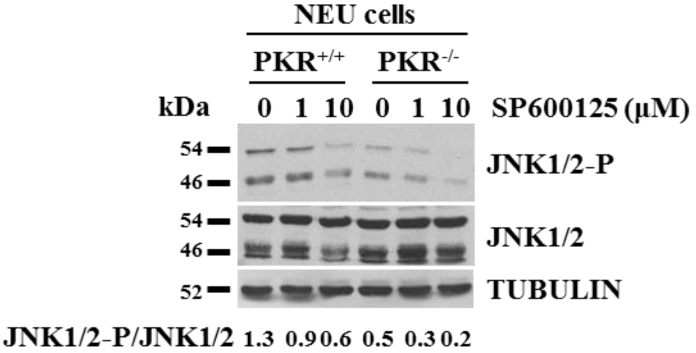

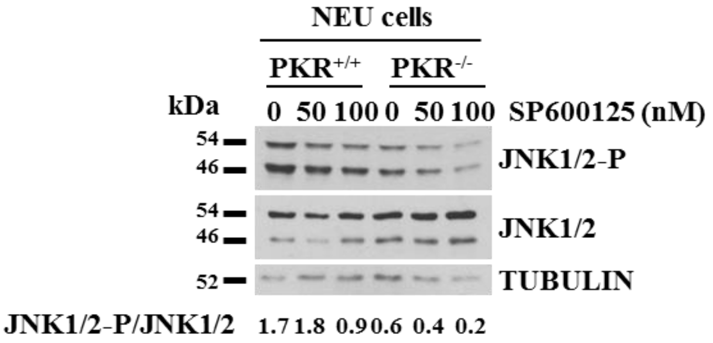

Supplementary Figure 4. Inhibition of JNK1/2-phowphorylation by SP600125. Mouse NEU PKR$^{+/+}$ and PKR$^{-/-}$ tumor cells were treated with indicated concentration of SP600125 and immunoblotted for phosphorylated and total JNK1/2 levels. Quantifications of the phosphorylated JNK1/2 normalized to total JNK1/2 for each lane are indicated.

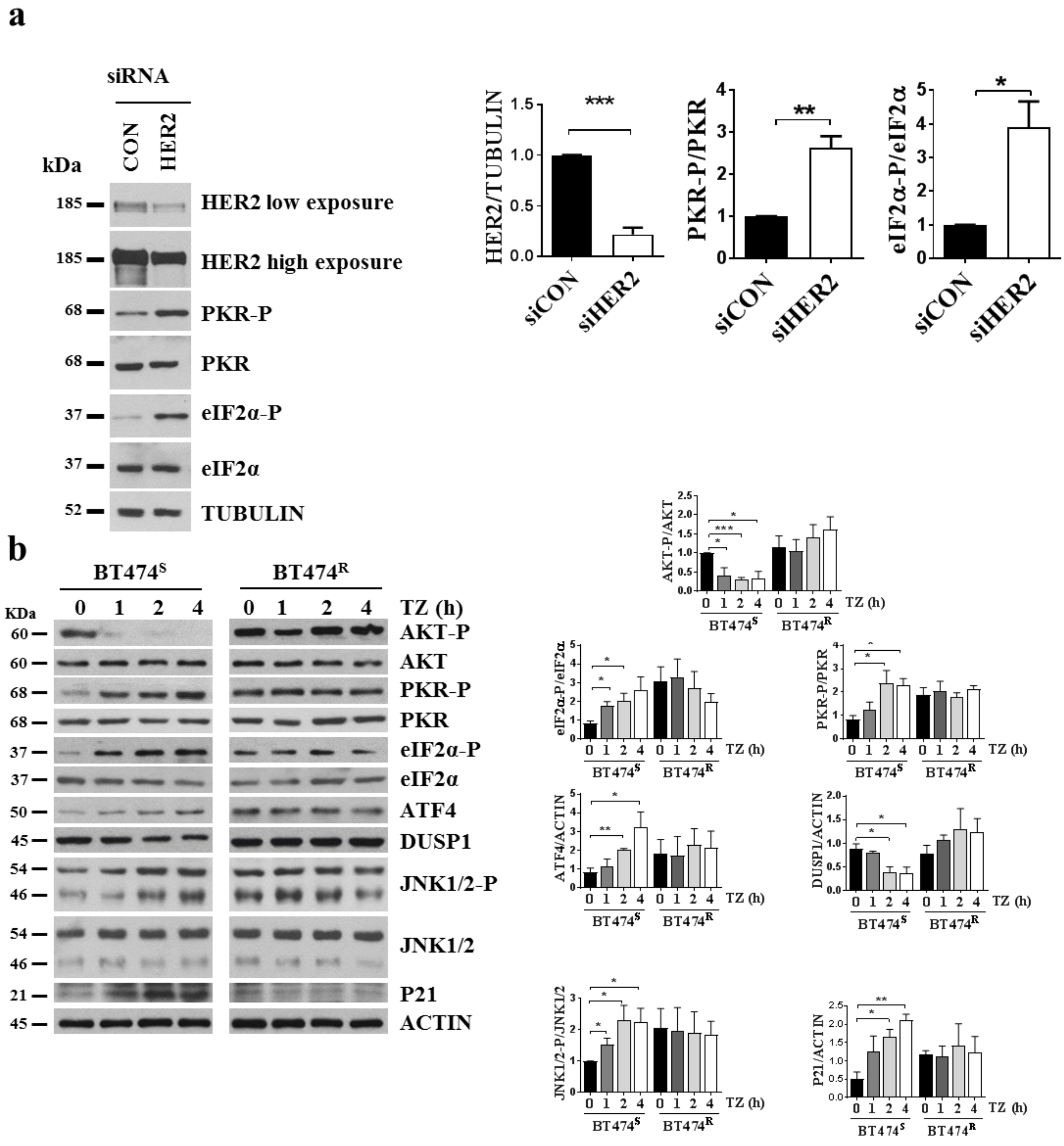

Supplementary Figure 5. PKR is inhibited by HER2 and activated by Trastuzumab in breast tumor cells. (**a**) BT474 cells were treated with scrambled siRNAs (CON) or HER2 siRNAs and subjected to immunoblotting for the indicated proteins. Quantification of blots from 3 biological replicates are indicated. (**b**) Immunoblot analyses of the indicated proteins in protein extracts from Trastuzumab sensitive (S) or resistant (R) BT474 cells treated with 21µg/ml Trastuzumab for the indicated time. Quantification of data represent from 3 biological replicates. (**a, b**) Data represent mean ±SEM *p < 0.05; **p<0.01; *** p<0.001

a

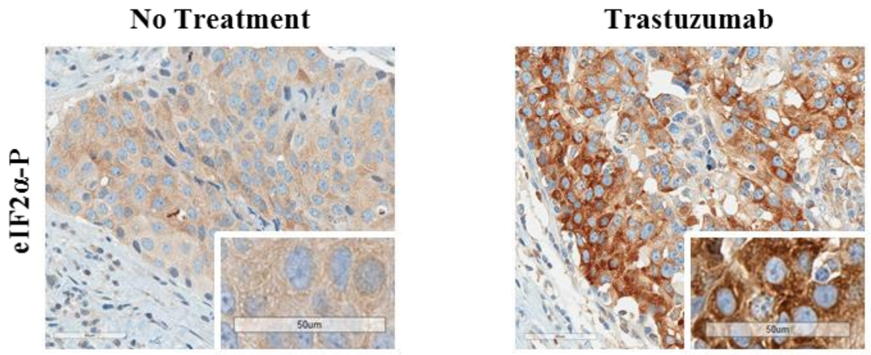

b

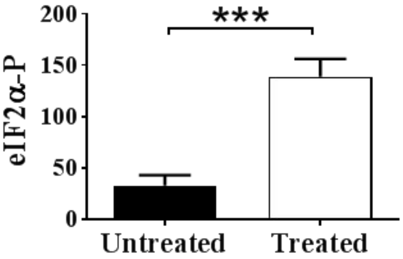

Supplementary Figure 6. Trastuzumab increases eIF2α-P in tumors from HER2+ breast cancer patients. (**a**) Representative IHC staining of eIF2α-P in HER2+ breast tumors before and after Trastuzumab treatment of the same patient, who developed resistance to therapy. (**b**) The intensity of eIF2α-P was evaluated by the Aperio ImageScope software. The graph shows the histoscore (H-score) of the intensity of eIF2α-P in tumors before and after Trastuzumab therapy of 6 patients of HER2+ breast cancer with resistance to Trastuzumab. Data represent mean ±SEM *** $p<0.001$

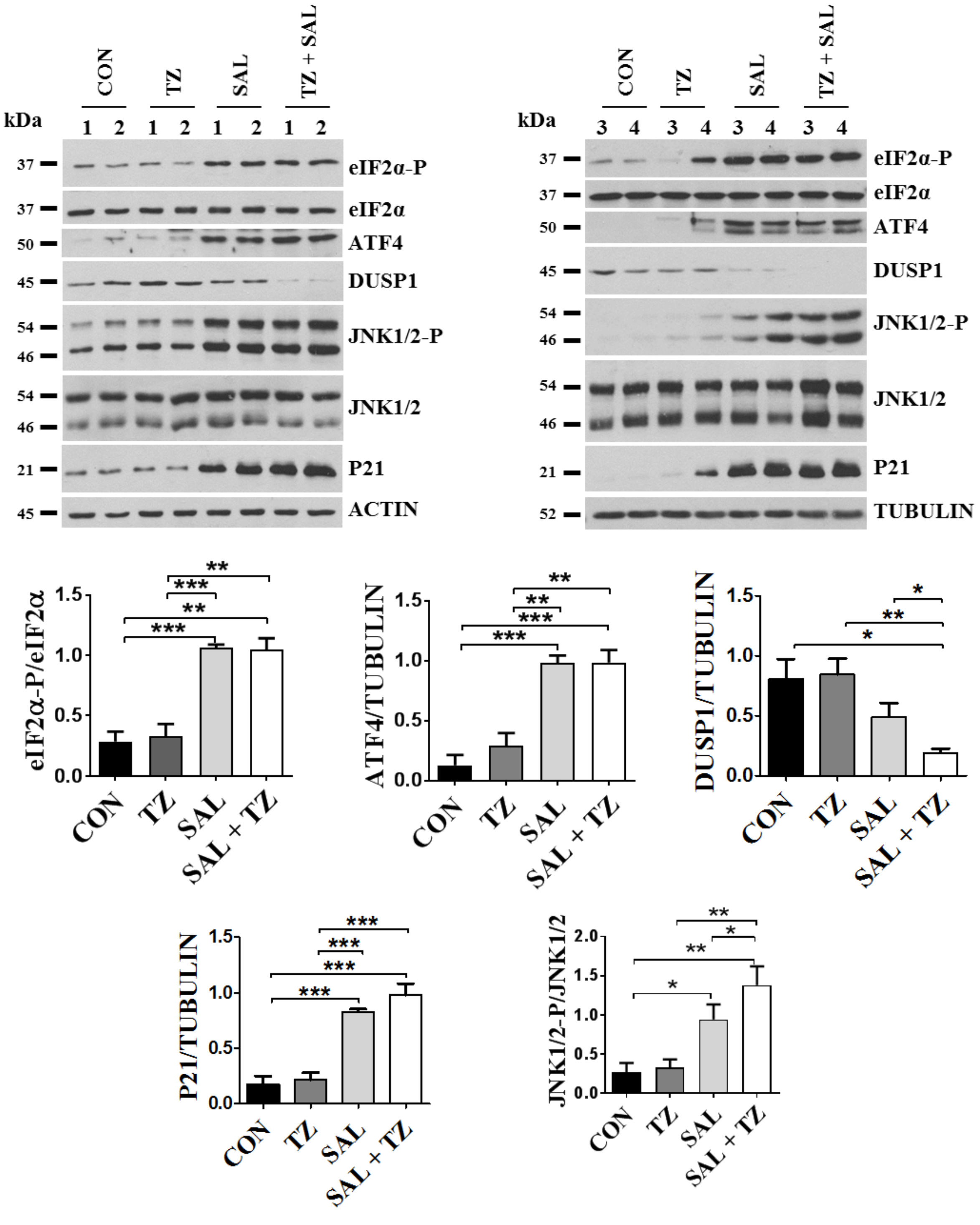

Supplementary Figure 7. SAL003 induces the anti-tumor pathways downstream of eIF2α-P in HER2+ gastric PDXs co-treated with Trastuzumab. Protein extracts of HER2+ gastric tumors grown in NOG mice treated with SAL003 and/or Trastuzumab in Fig. 6 were subjected to immunoblotting for the indicated proteins. Quantifications were performed with data from the analyses of 4 different tumors per type of treatment. Data represent mean ±SEM $*p < 0.05$; $**p<0.01$; $***p<0.001$.

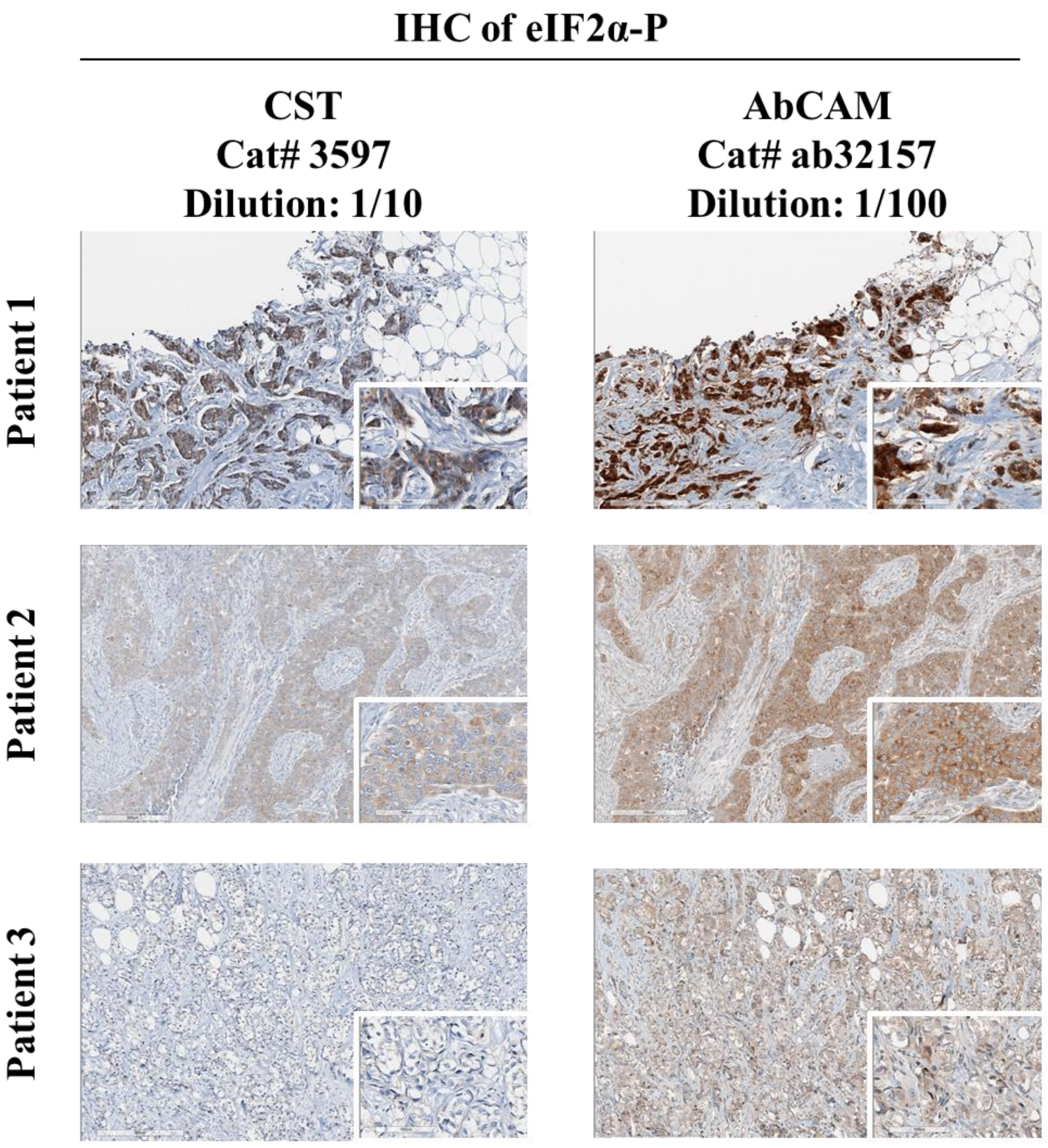

Supplementary Figure 8. Quality control analysis of the anti-eIF2α-P antibody. IHC analyses of identical tumor sections from 3 Trastuzumab-resistant HER2+ breast cancer patients, which were included in Supplementary Fig. 6, with an anti-eIF2α-P antibody from Cell Signaling Technology (CST Cat# 3597) or Abcam (Cat# ab32157) used in this study.

Application	Target gene	Sequence
**gRNA**	PKR	5'-TAATGGCTACTCCGTGCATC-3'
**shRNA**	DUSP1	#1: 5'-CCGGCATGAGGACTAACCGGGTAAACTCGAGTTTACCCGGTTAGTCCTCATGTTTTT-3'
		#2: 5'-CCGGGTTGTTGGATTGTCGCTCCTTCTCGAGAAGGAGCGACAATCCAACAACTTTTT-3'
	ATF4	#1: 5'-CCGGCCAGAGCATTCCTTTAGTTTACTCGAGTAAACTAAAGGAATGCTCTGGTTTTTG-3'
		#2: 5'-CCGGCGGACAAAGATACCTTCGAGTCTCGAGACTCGAAGGTATCTTTGTCCGTTTTTG-3'
**RT-PCR**	DUSP1	Fw 5'-GTTGTTGGATTGTCGCTCCTTC-3'
		Rv 5'-TTGGGCACGATATGCTCCAG-3'
	P21	Fw 5'-GCAGATCCACAGCGATATCC-3'
		Rv 5'-CAACTGCTCACTGTCCACGG-3'
	ACTIN	Fw 5'-CAGCAGATGTGGATCAGCAAG-3'
		Rv 5'-GCATTTGCGGTGGACGAT-3'
	GAPDH	Fw 5'-GAGAGTGTTTCCTCGTCCCG-3'
		Rv 5'-CAATCTCCACTTTGCCACTGC-3'

Supplementary Table 1. Sequences of DNA primers used in the study.

Antibody	Species	Company	Cat #	Dilution WB	IHC
phosphoserine 51-eIF2α	rabbit monoclonal	Novus Biologicals	NB 110-56949	1/1000	1/100
phosphoserine 51-eIF2α	rabbit monoclonal	Cell Signaling	3597		1/10
phosphoserine 51-eIF2α	rabbit monoclonal	AbCAM	ab32157		1/100
phosphothreonine 446-PKR	rabbit monoclonal	AbCAM	ab32036	1/1000	
PKR	mouse monoclonal	Santa Cruz	sc-6282	1/1000	
ATF4	rabbit polyclonal	Proteintech	10835-1-AP	1/1000	
P21 Waf/Cip1 (12D1)	rabbit monoclonal	Cell Signaling	2947	1/1000	
Actin	mouse monoclonal	mpbio	C4	1/5000	
phospho-Akt (Ser473)	rabbit monoclonal	Cell Signaling	4060	1/1000	
Akt	rabbit polyclonal	Cell Signaling	9272	1/1000	
DUSP1	rabbit polyclonal	Millipore	7535	1/1000	
HER2	rabbit polyclonal	Cell Signaling	2165	1/1000	
eIF2α	mouse monoclonal	Cell Signaling	2103	1/1000	
JNK1/2-P	rabbit monoclonal	Cell Signaling	4668	1/1000	
JNK1/2	rabbit polyclonal	Cell Signaling	9252	1/1000	
α-Tubulin	mouse monoclonal	Sigma-aldrich	T5168	1/1000	
Ki67	rabbit polyclonal	AbCAM	ab15580		1/500
Cleaved Caspase-3 (Asp175)	rabbit polyclonal	Cell Signaling	9661		1/150
Mouse IgG-horseradish peroxidase-conjugated	goat	KPL	474-1806	1/3000	
Rabbit IgG-horseradish peroxidase-conjugated	goat	Jackson immunoReseach	111-035-144	1/3000	
Biotinylated anti-rabbit IgG	horse	Vector	BA-1100		1/150

Supplementary Table 2. Primary and secondary antibodies used in the study.

Blots of Figure 1d

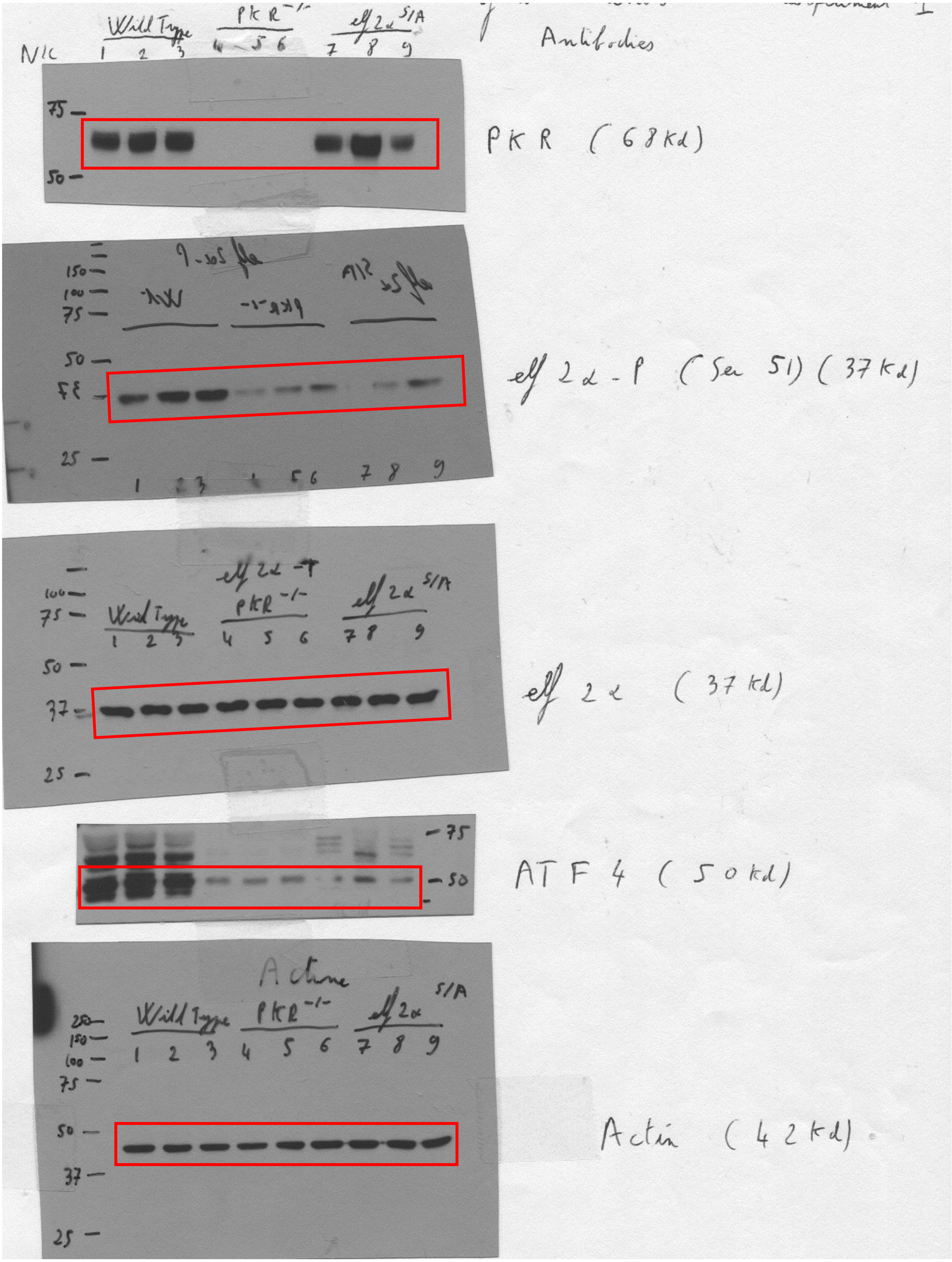

Blots of Figure 2d

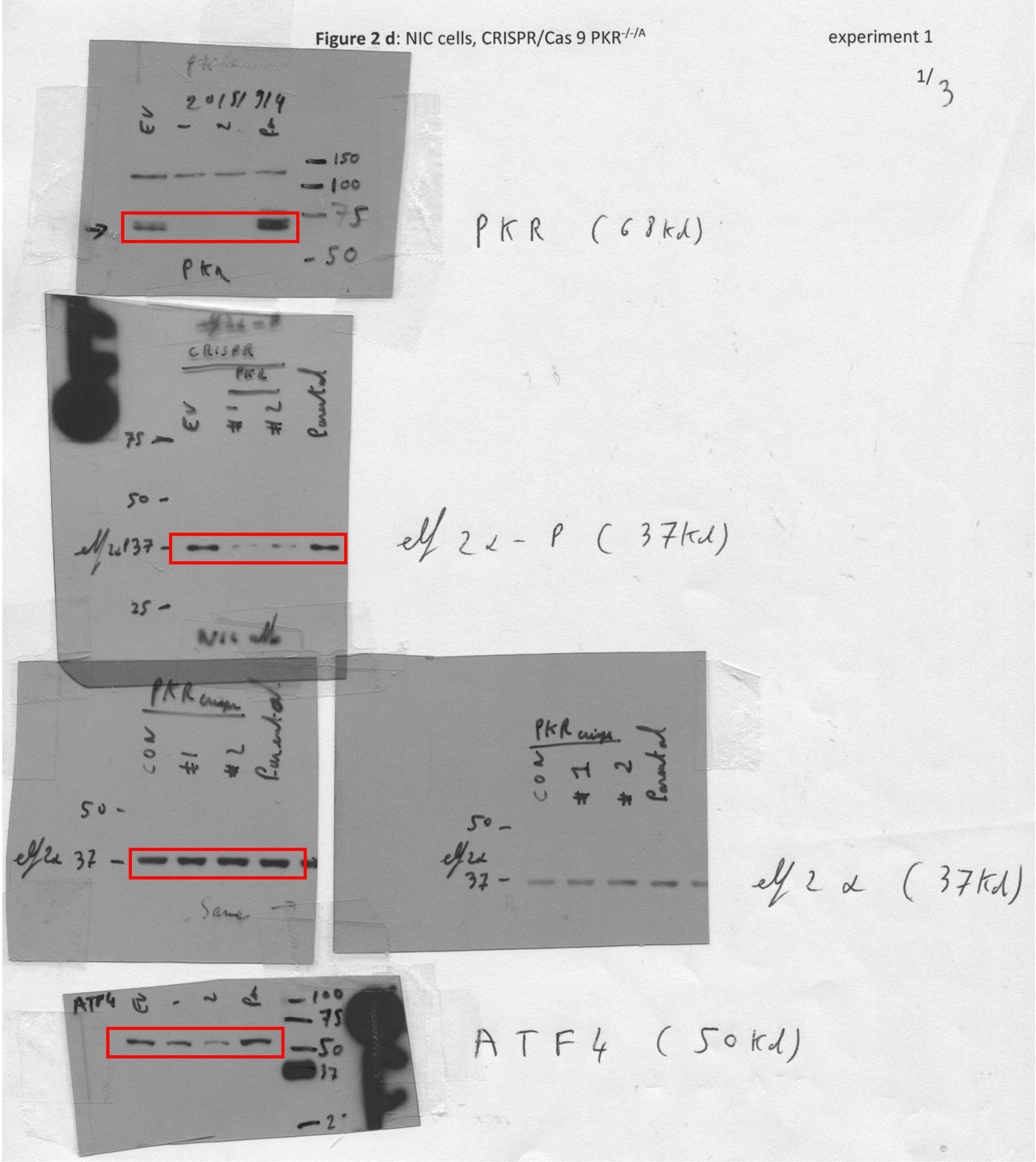

Blots of Figure 2d (cont'd)

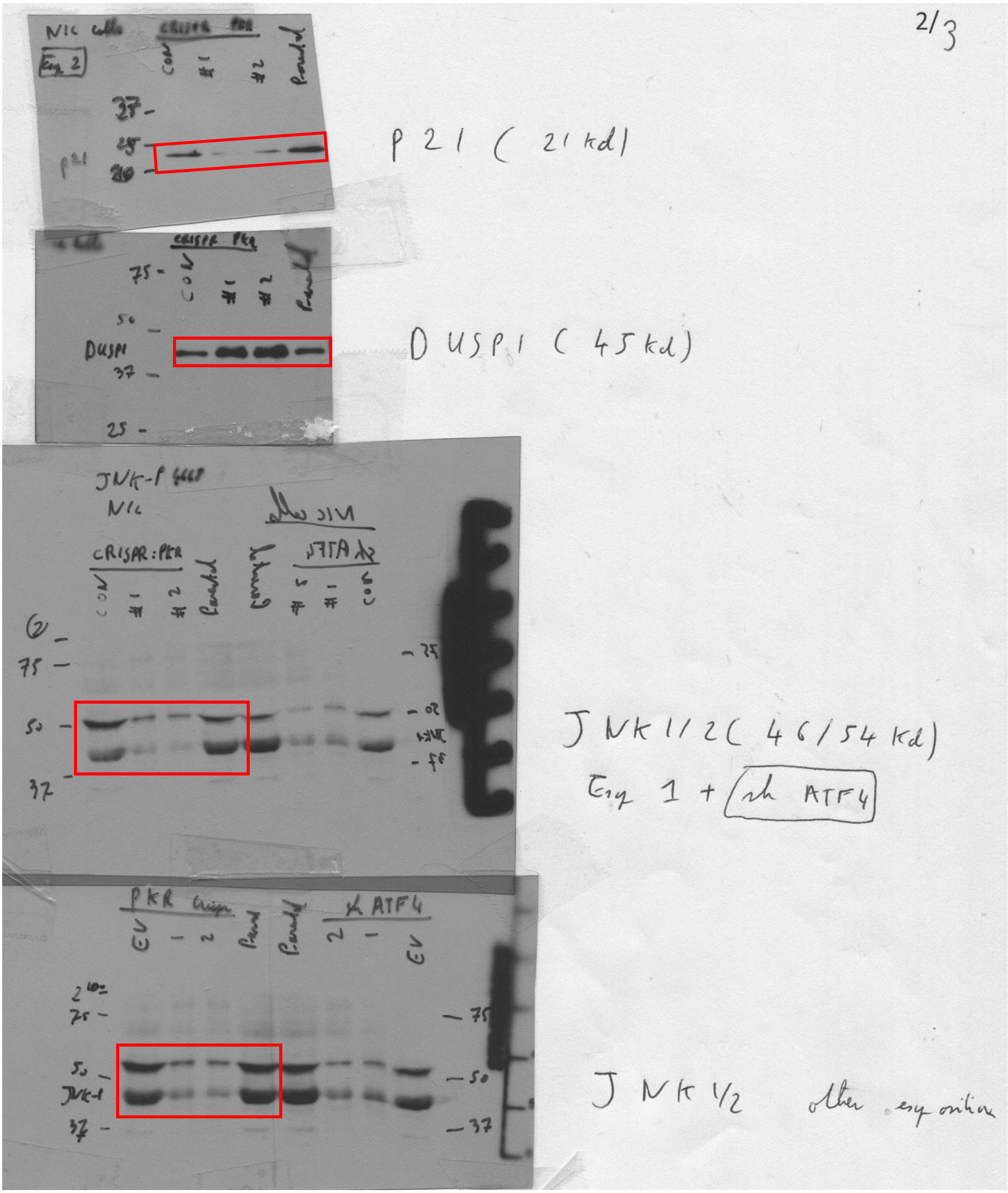

Blots of Figure 2d (cont’d)

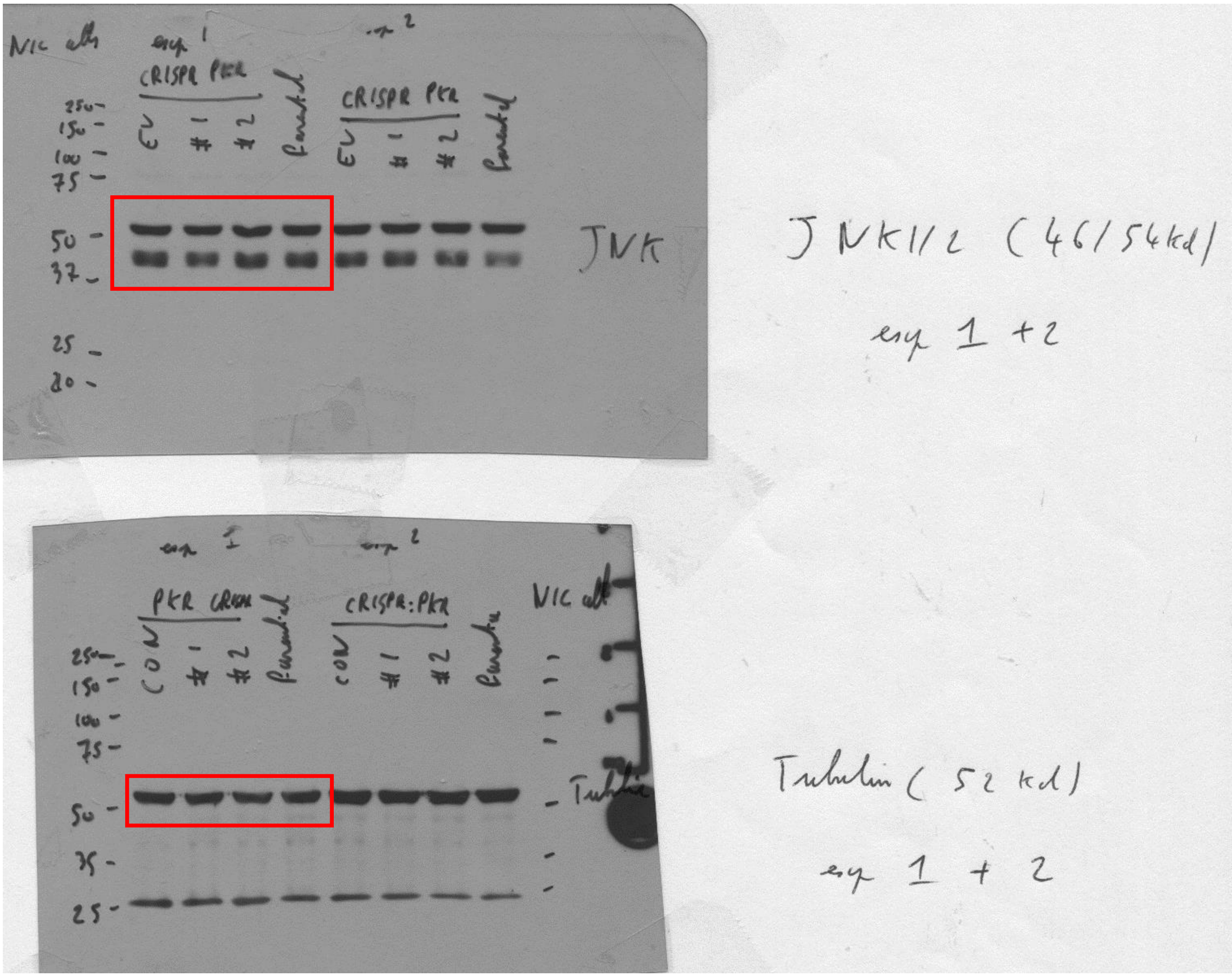

Blots of Figure 3a

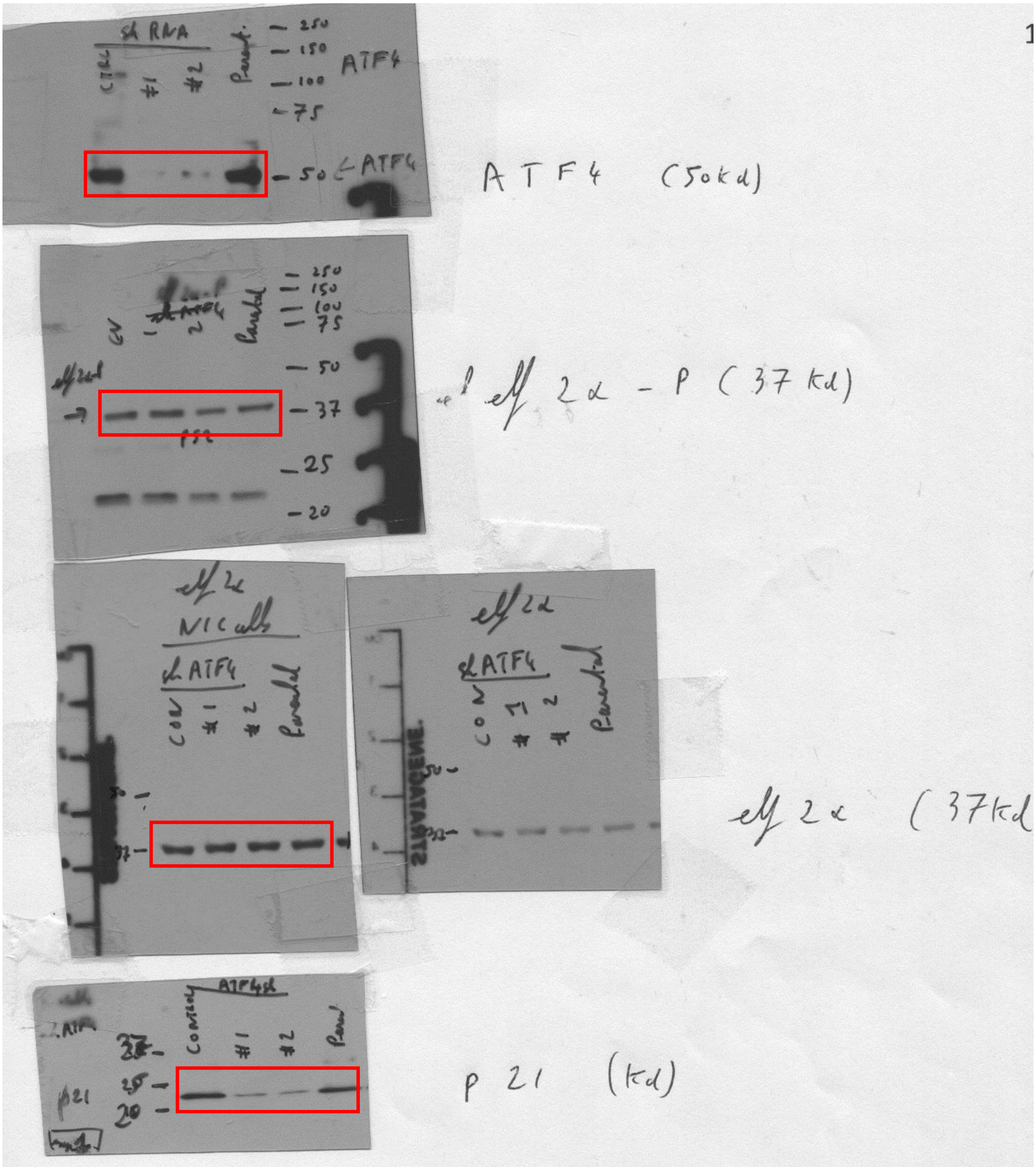

Blots of Figure 3a (cont'd)

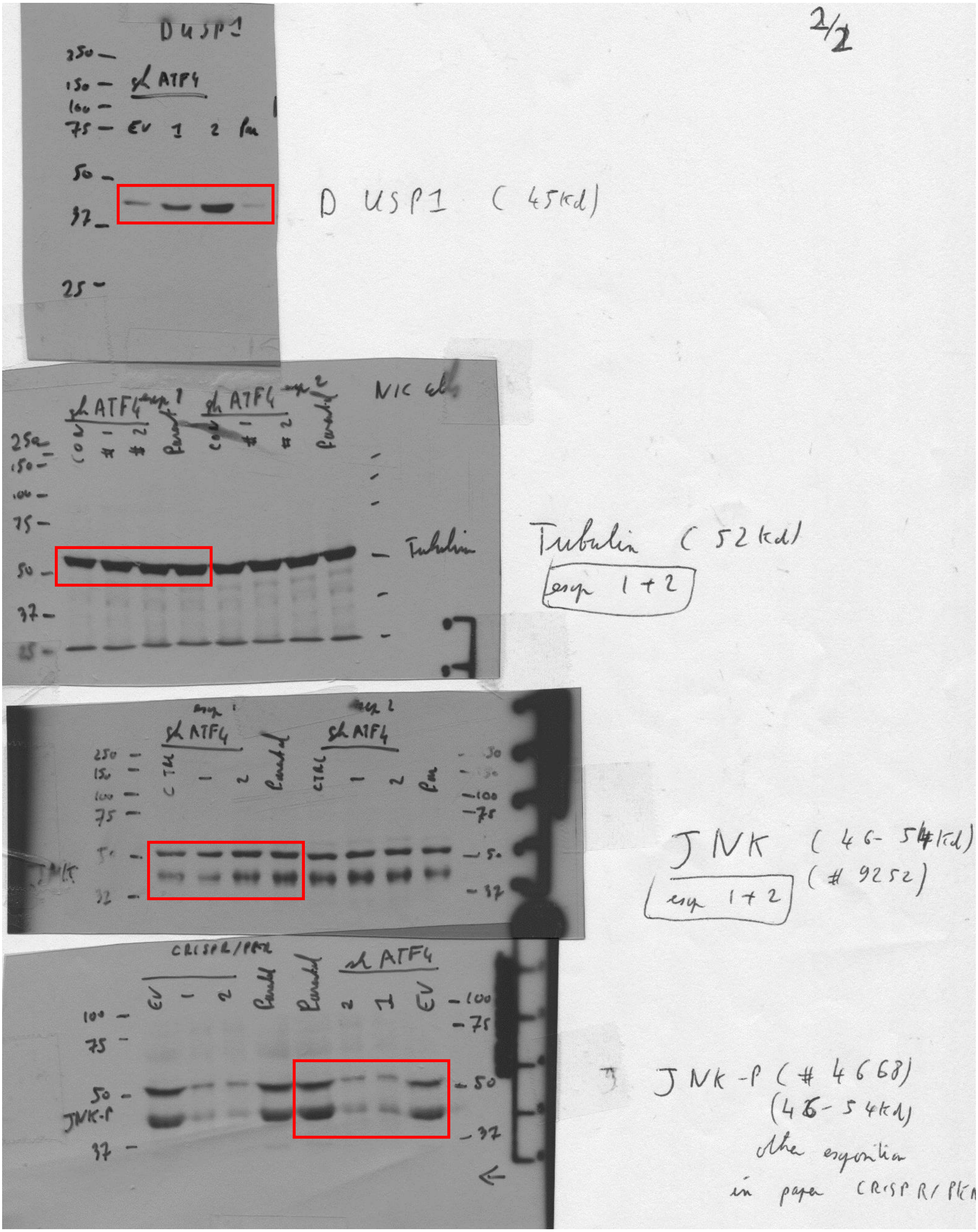

Blots of Figure 4a

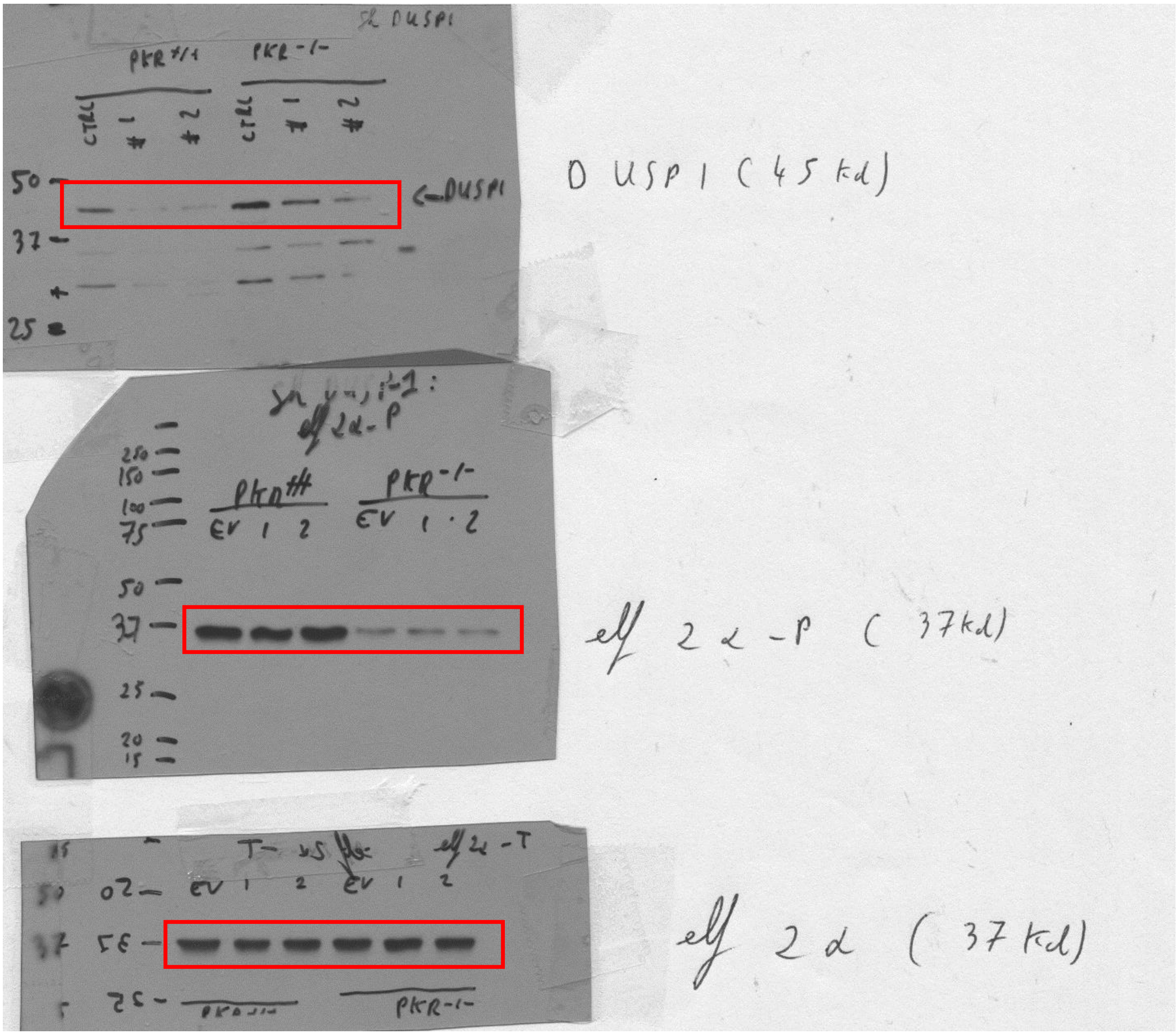

Blots of Figure 4a (cont'd)

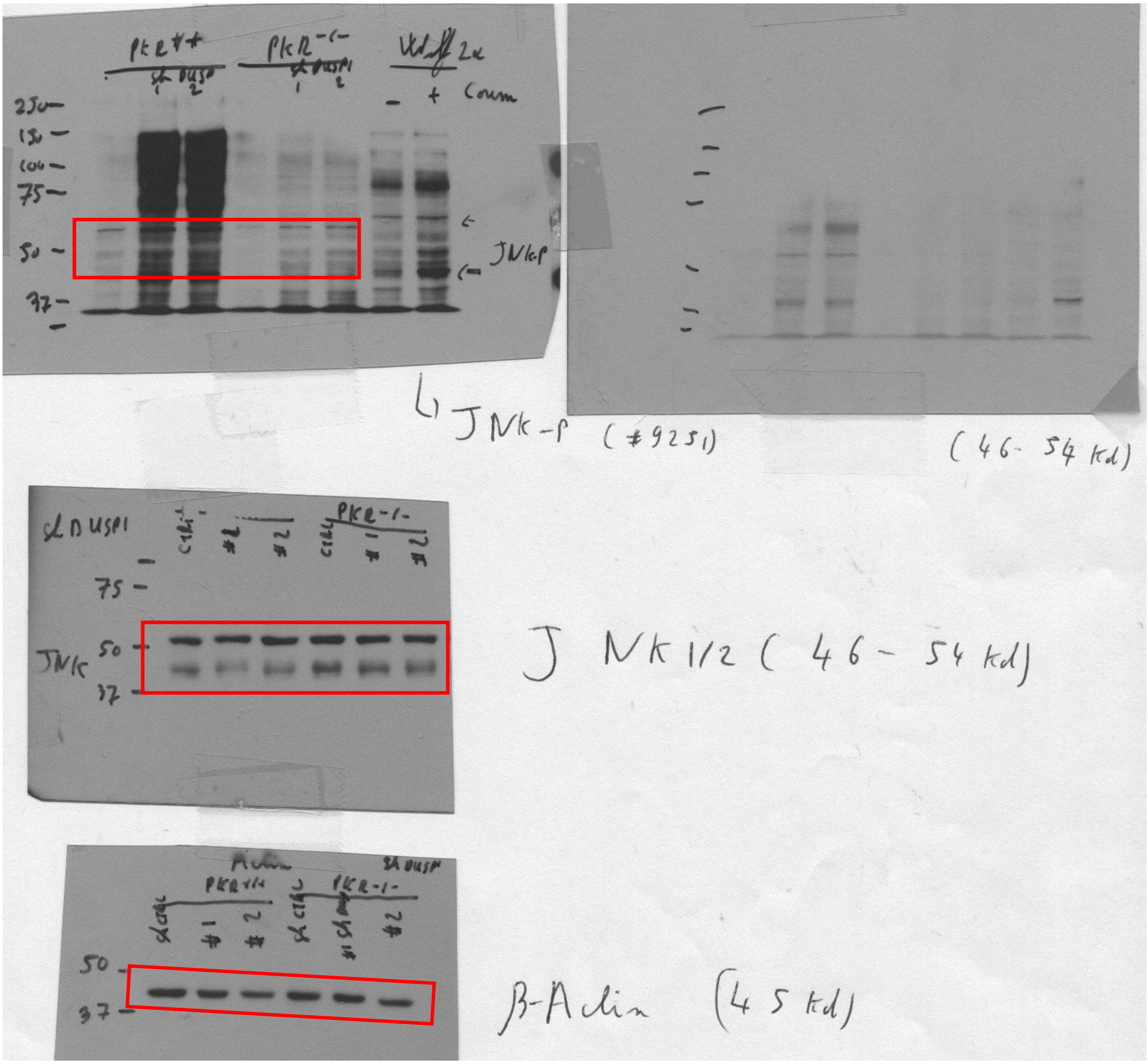

Blots of Figure 5a

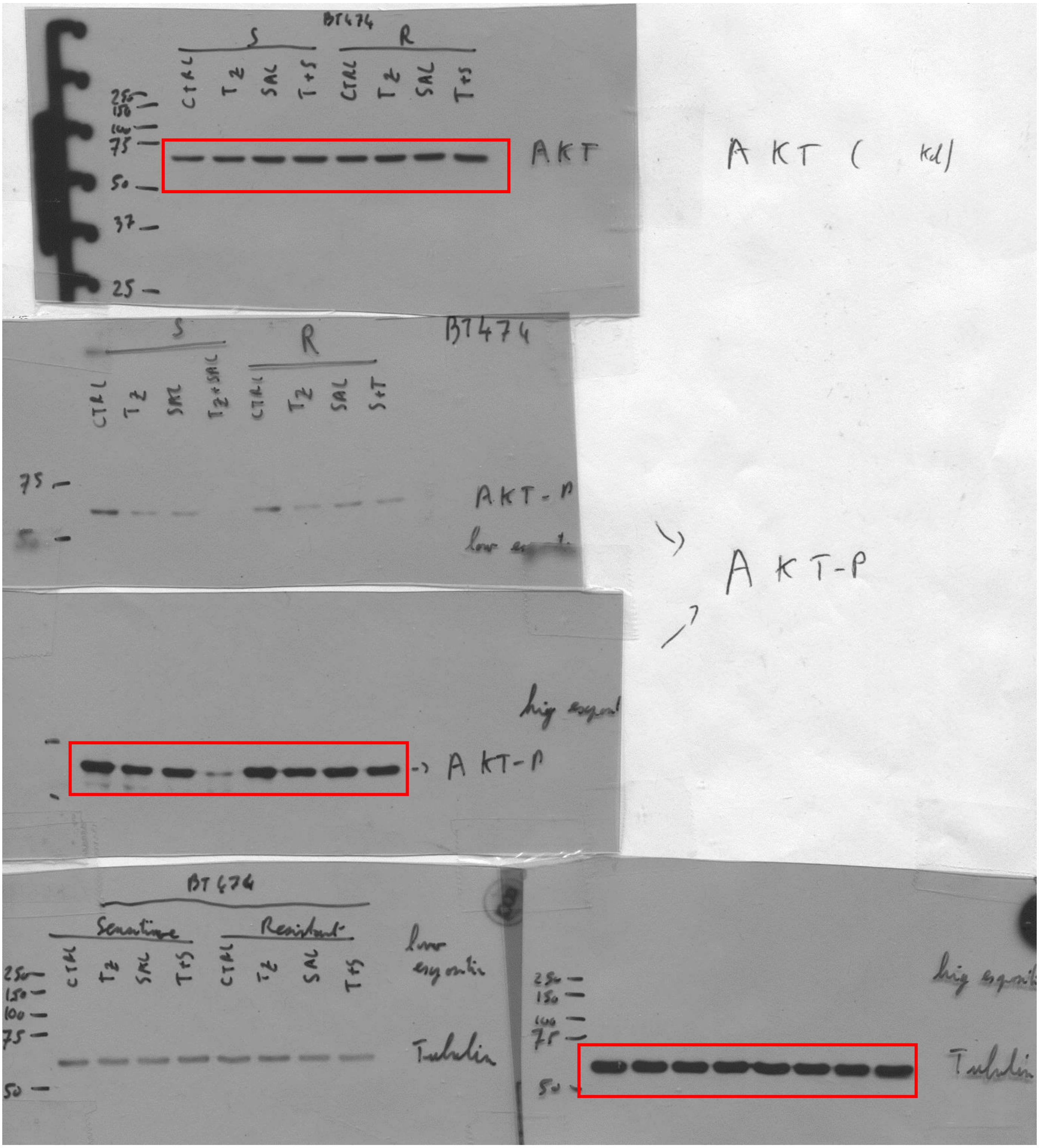

Blots of Figure 5a (cont'd)

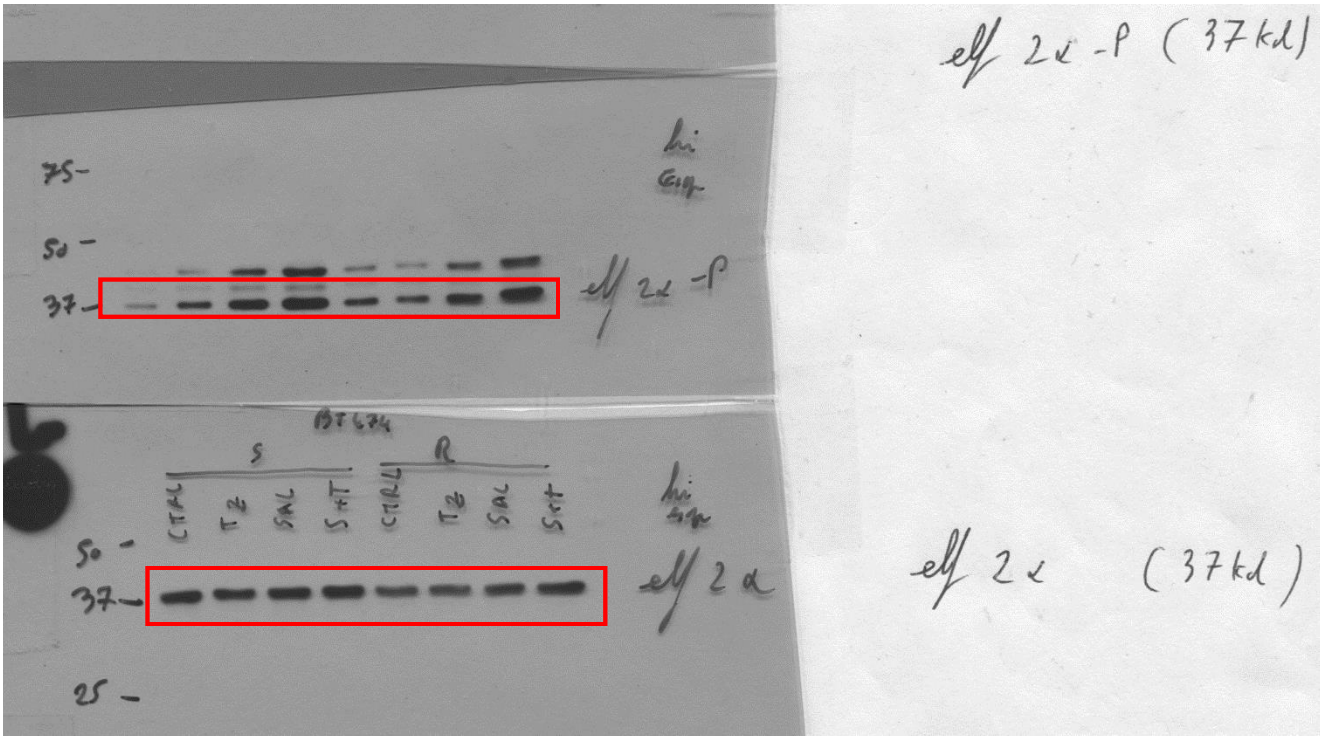

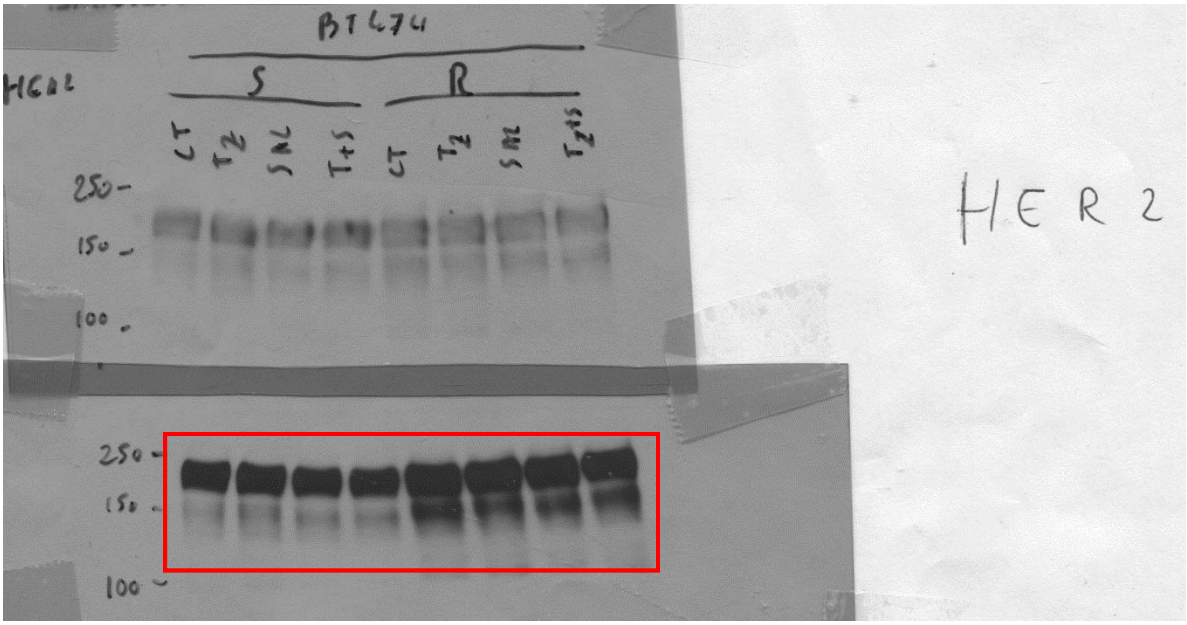

Blots of Figure 5a (cont'd)

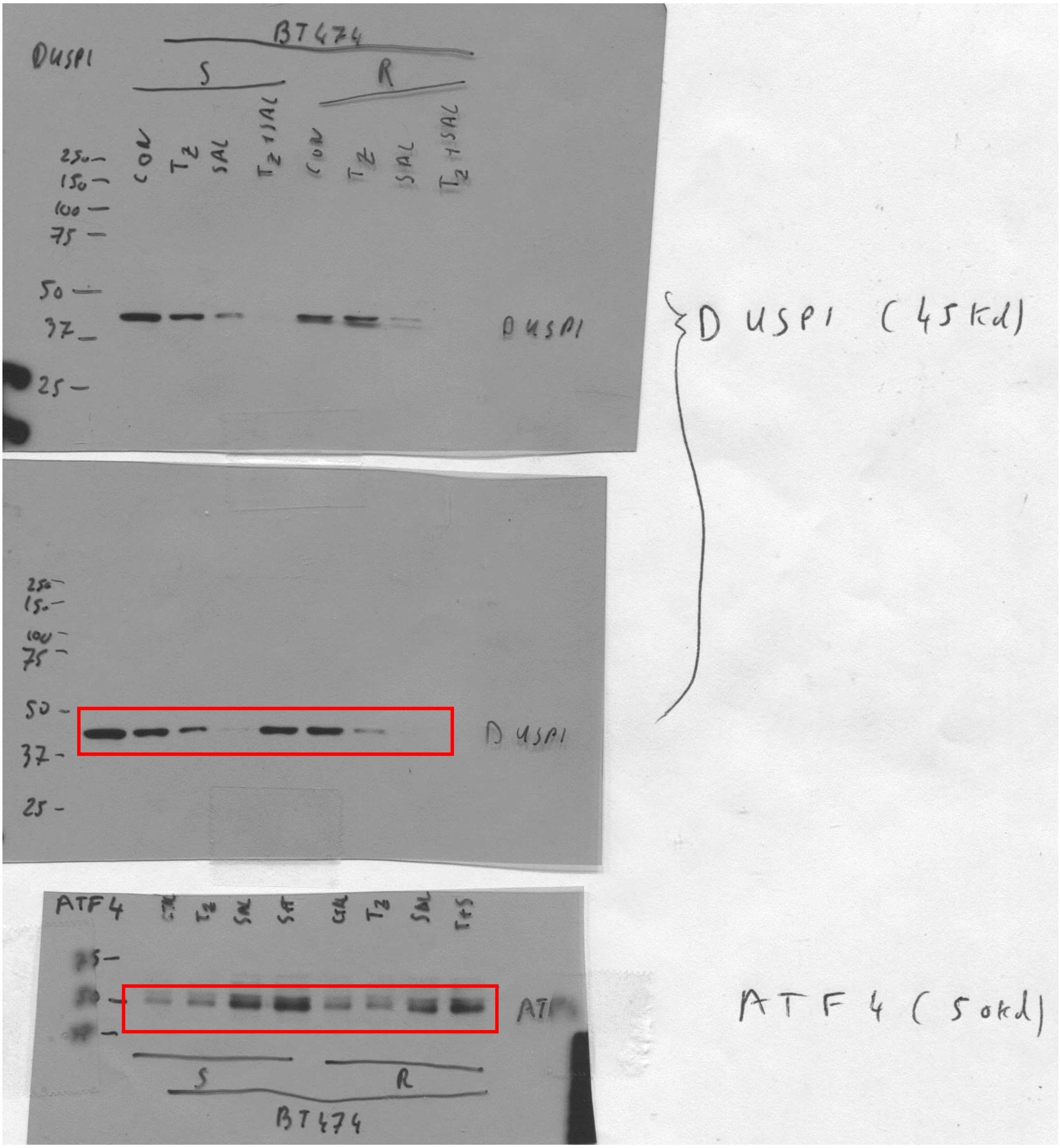

Blots of Figure 5a (cont'd)

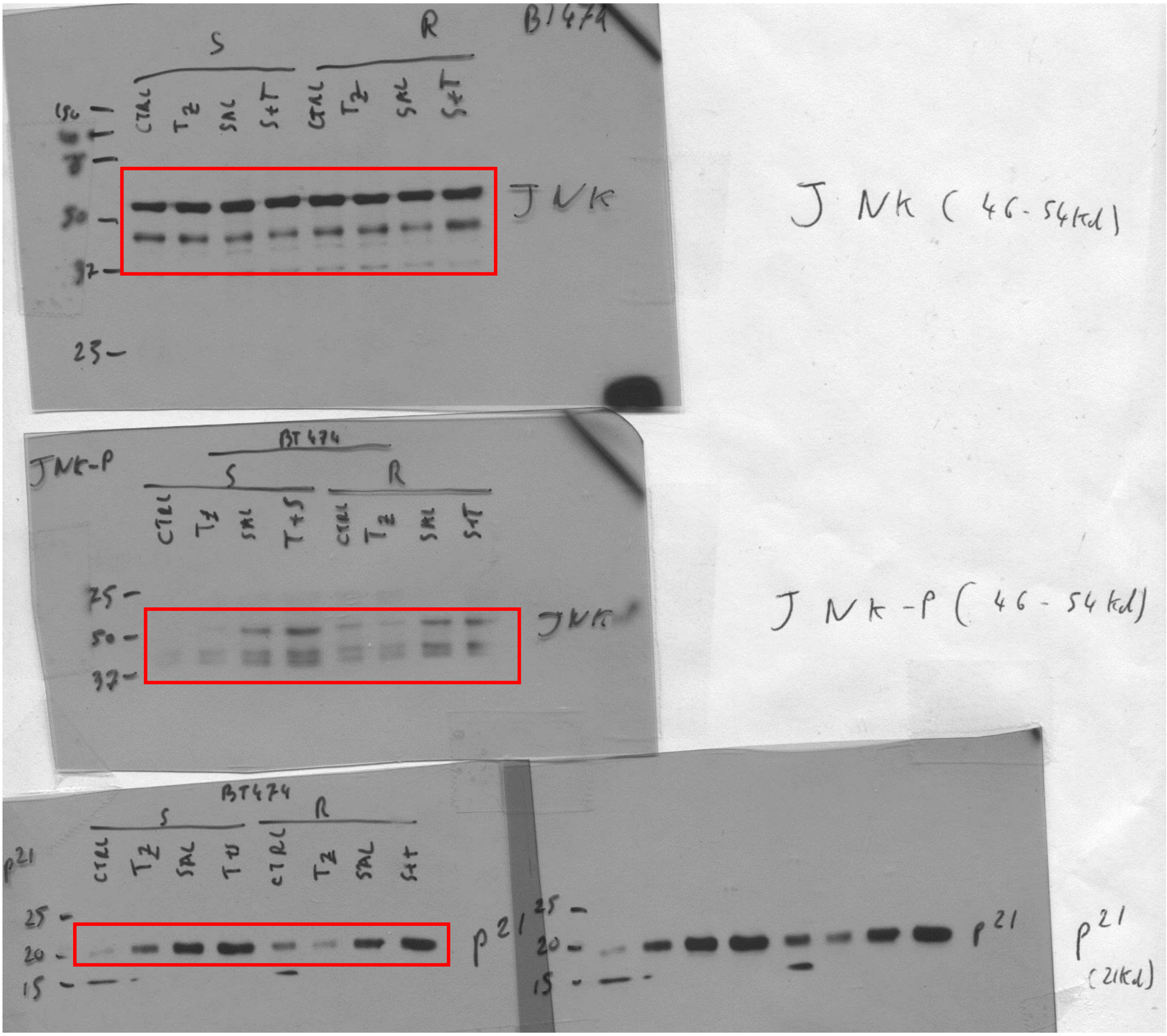

Blots of Supplementary Figure 1

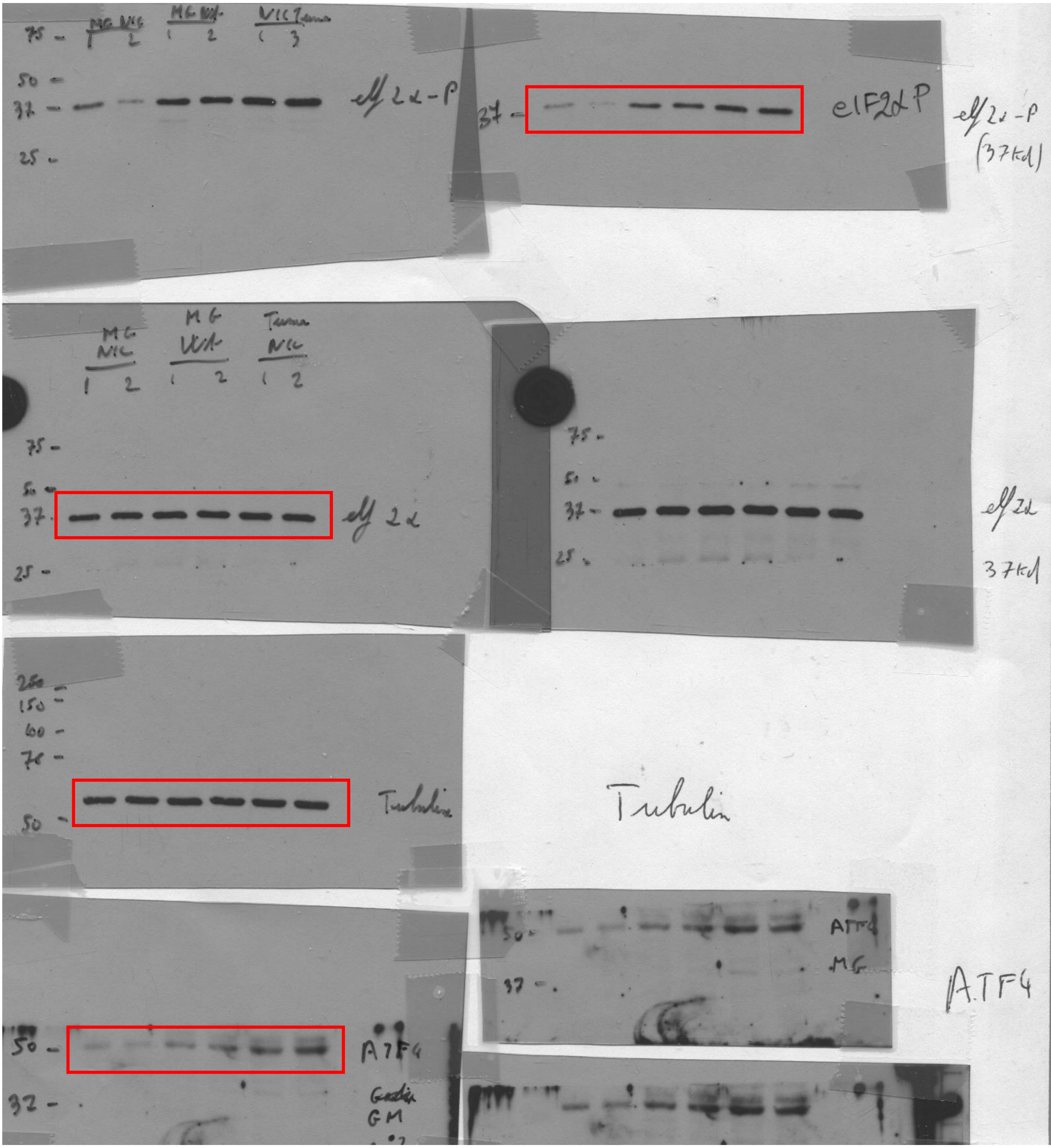

Blots of Supplementary Figure 1 (cont'd)

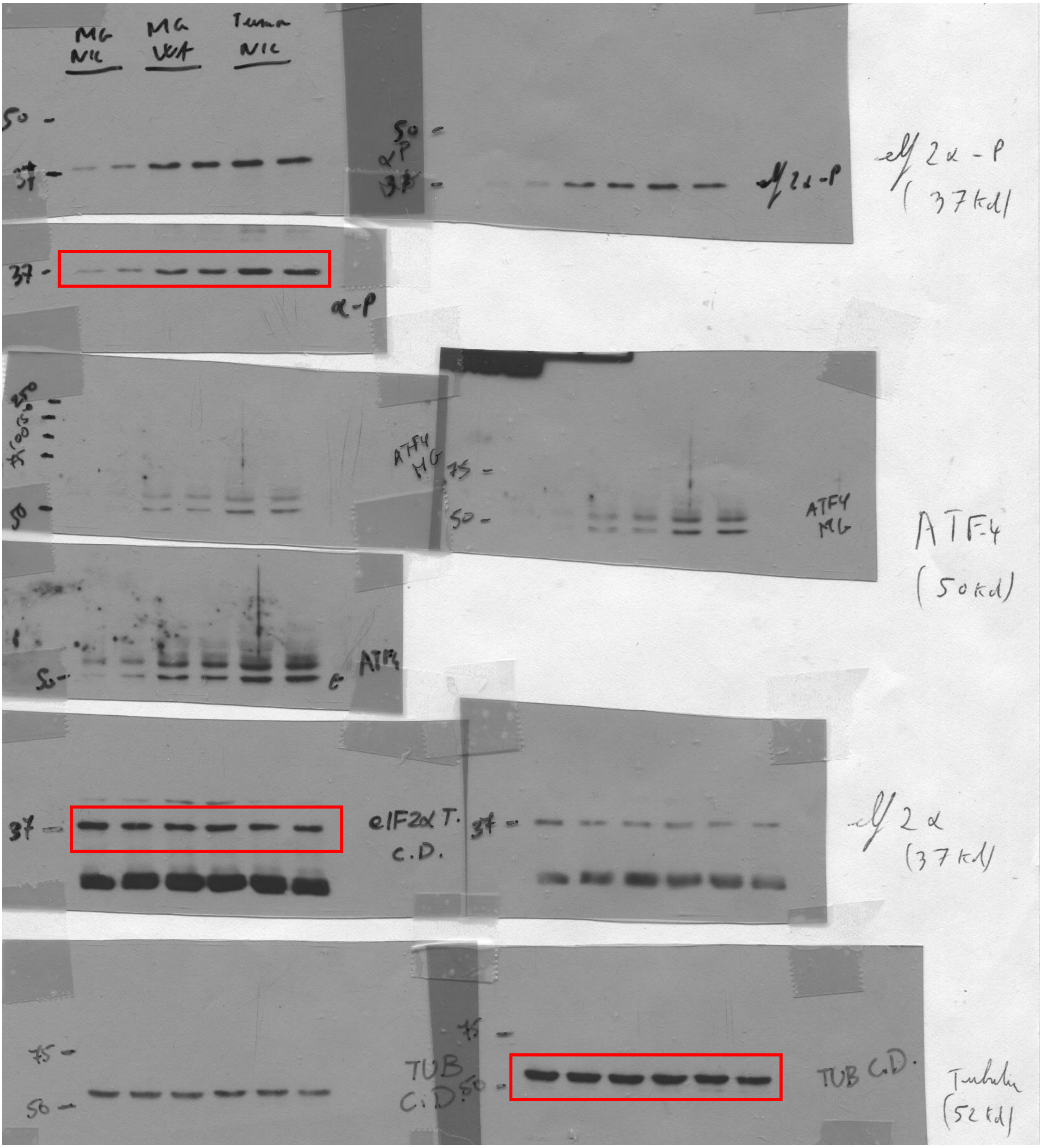

Blots of Supplementary Figure 4

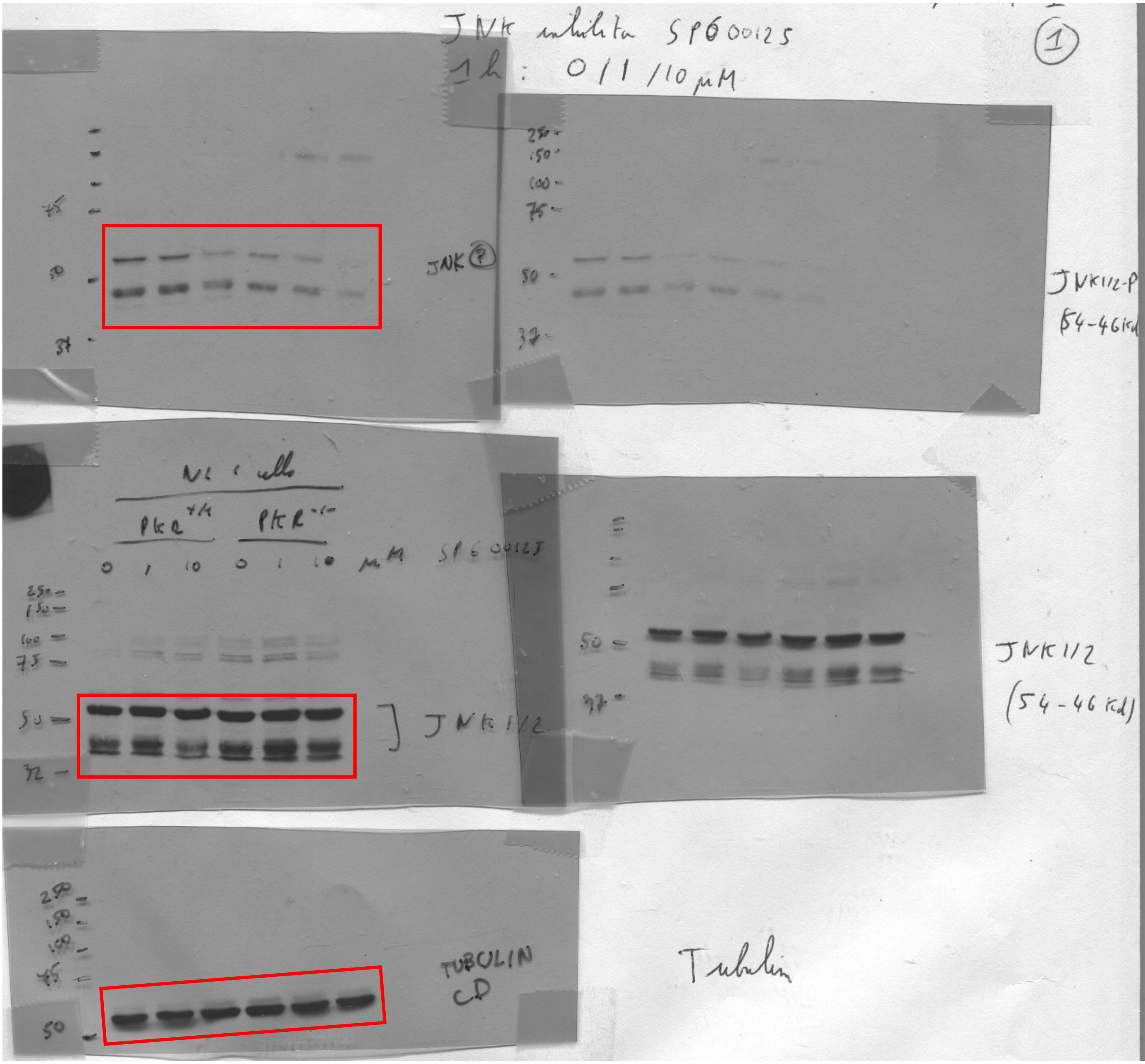

Blots of Supplementary Figure 4 (cont'd)

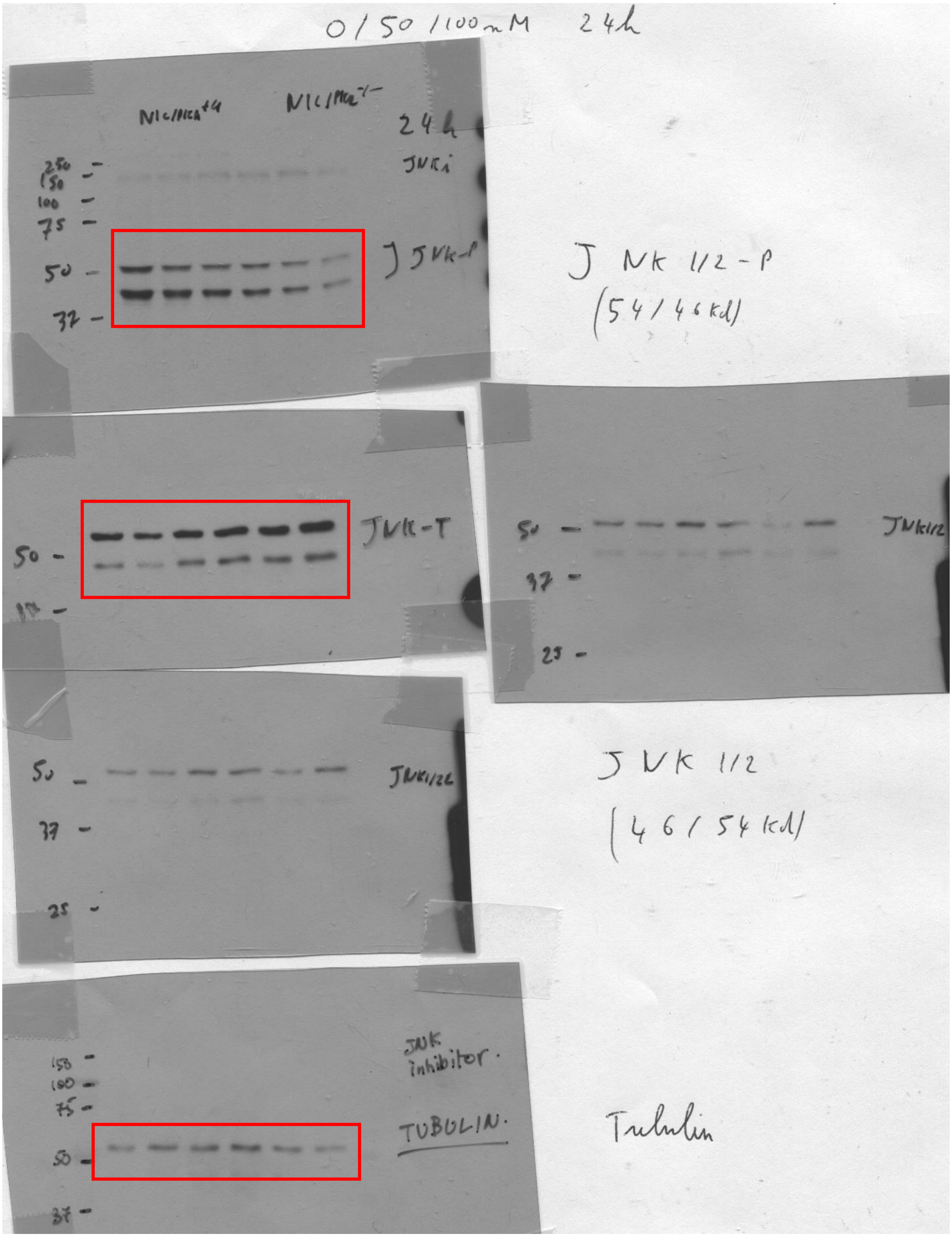

Blots of Supplementary Figure 5a

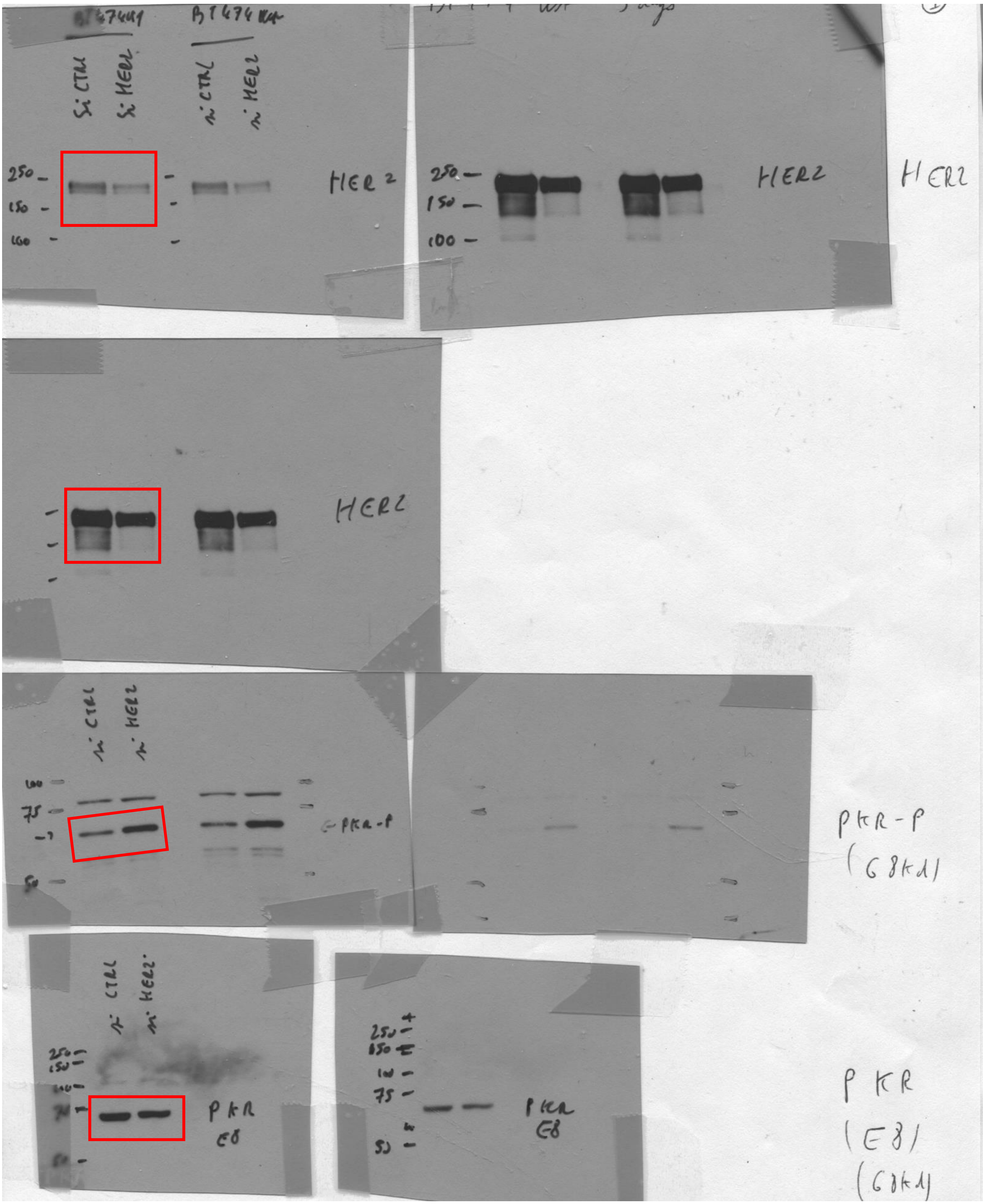

Blots of Supplementary Figure 5a (cont'd)

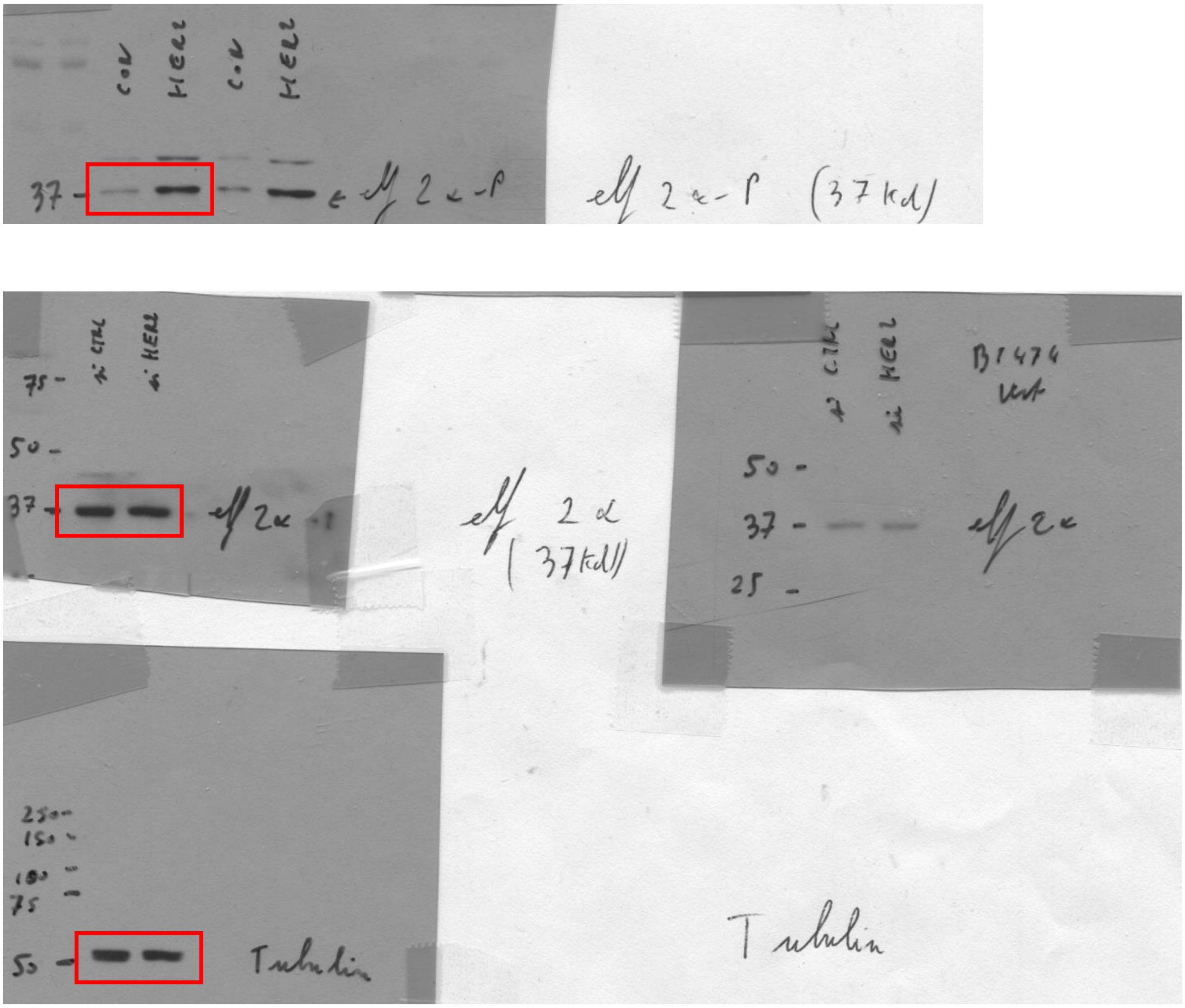

Blots of Supplementary Figure 5b

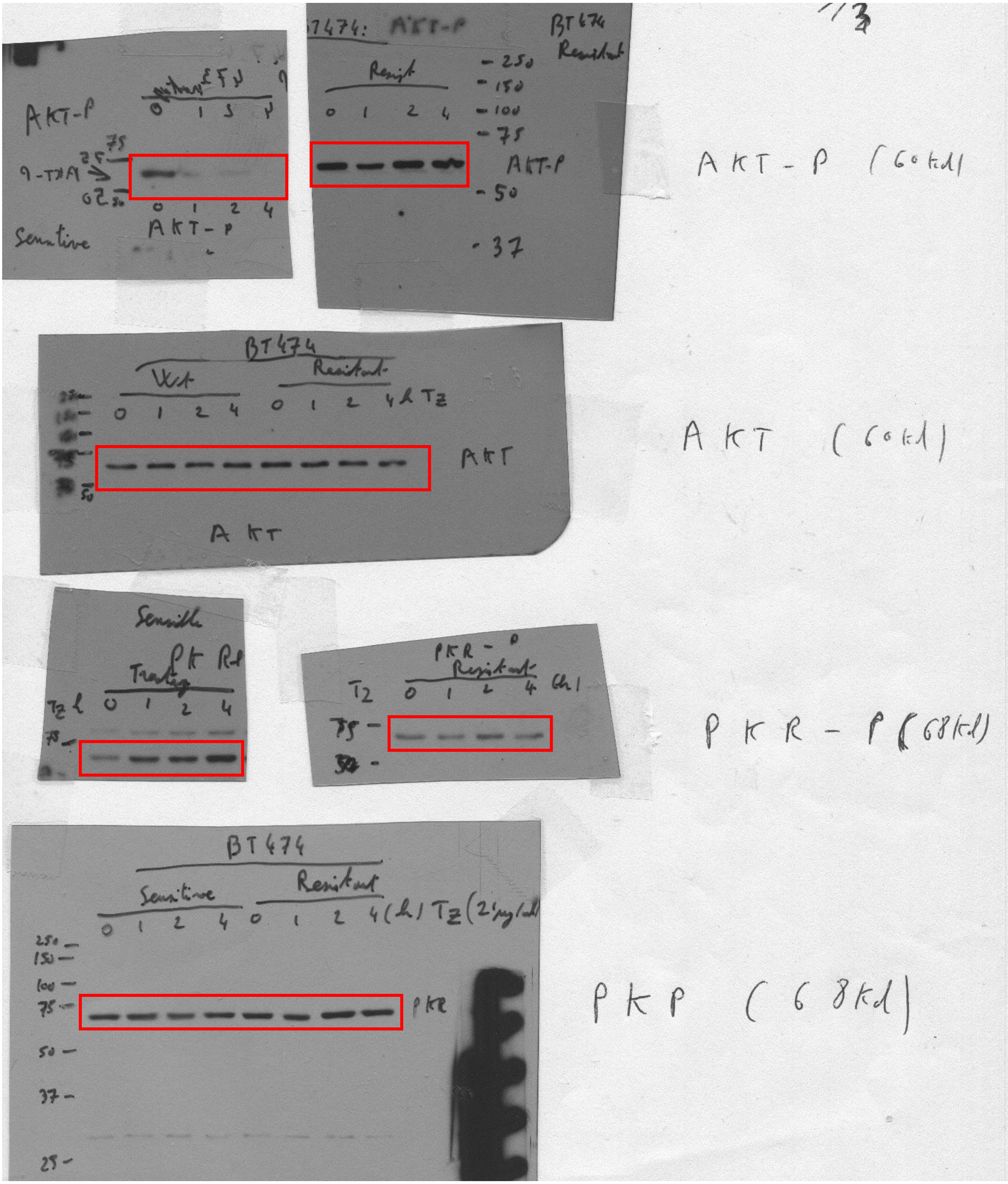

Blot of Supplementary Figure 5b (cont'd)

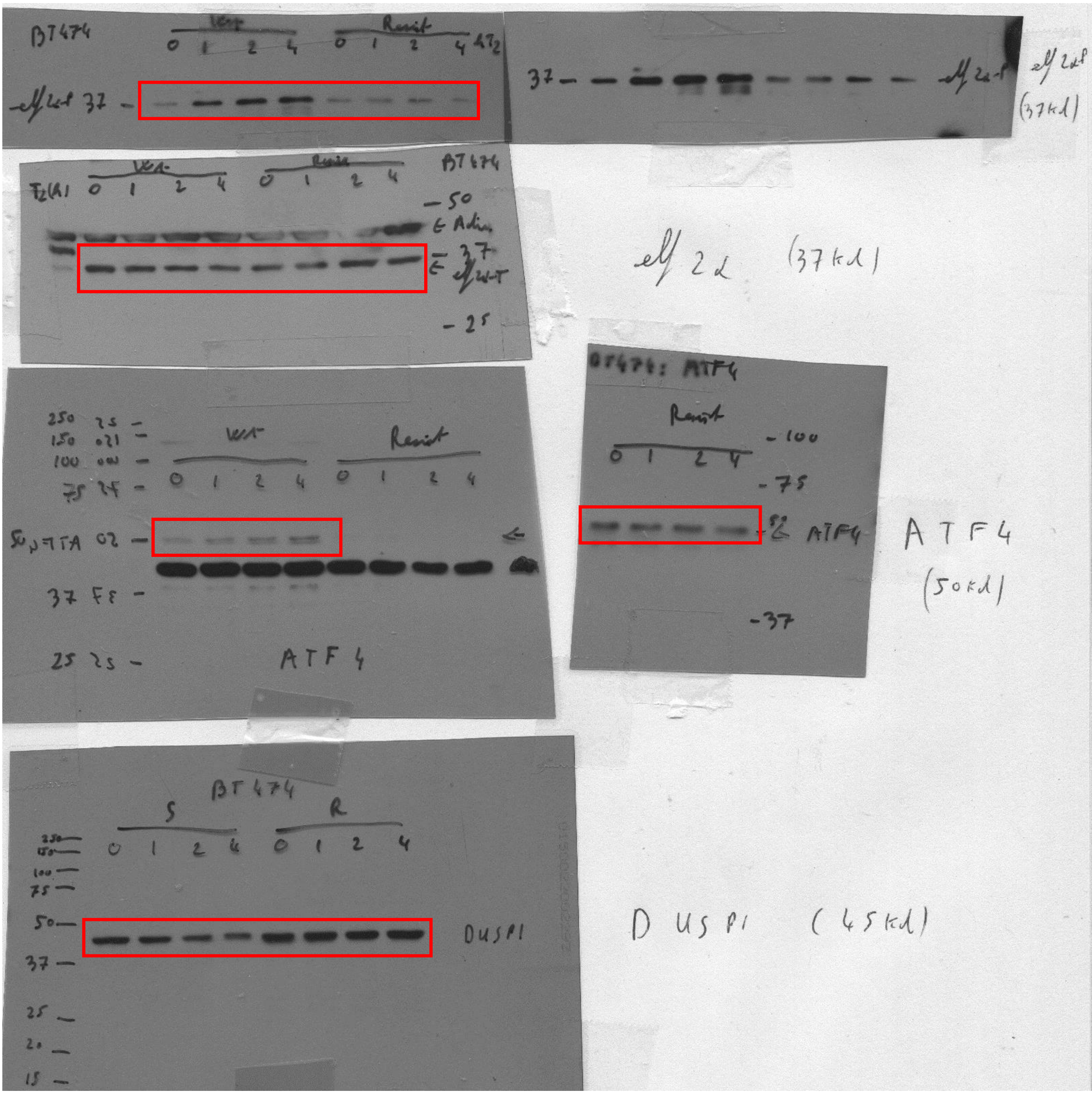

Blots of Supplementary Figure 5b (cont'd)

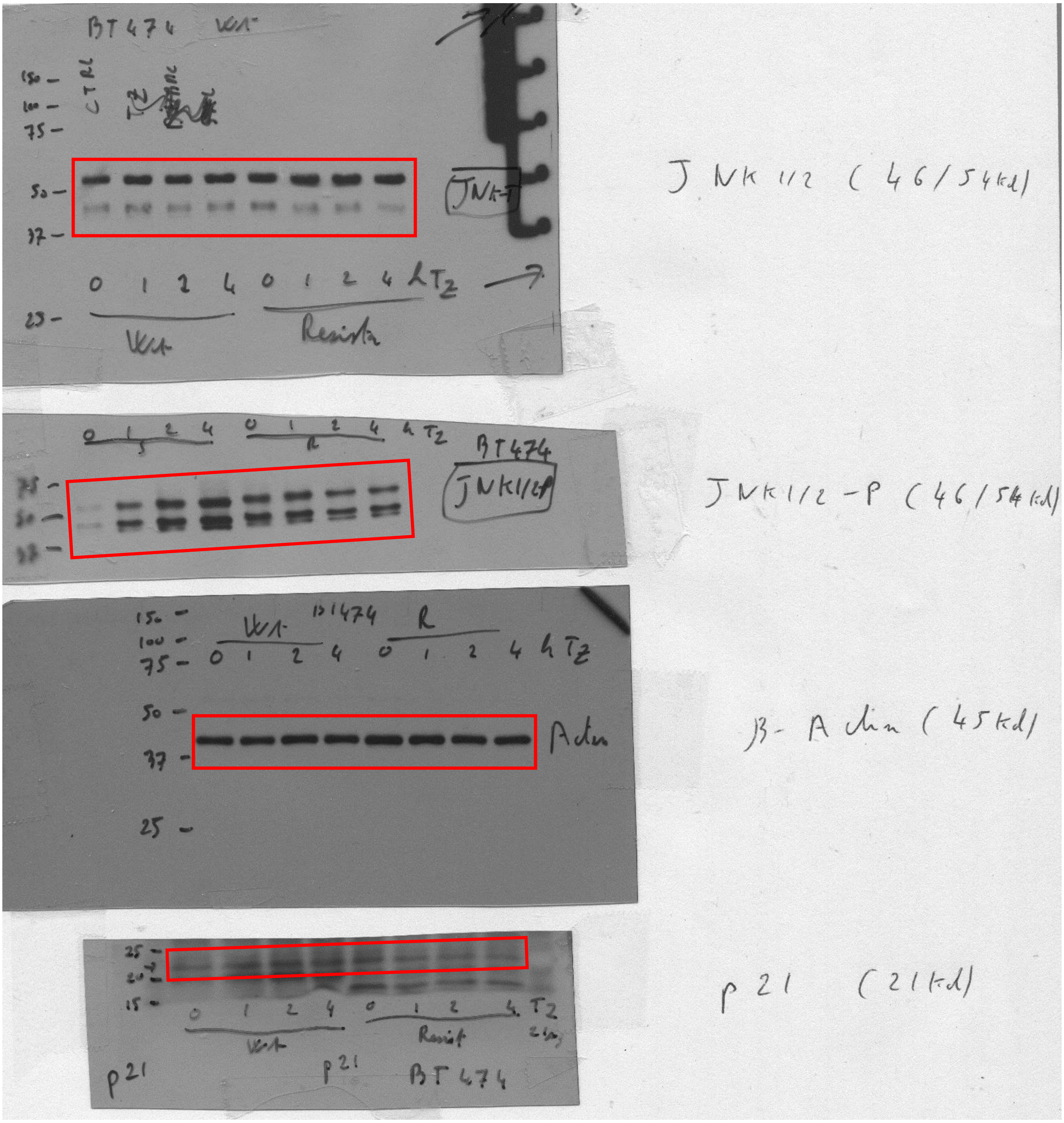

Blots of Supplementary Figure 7

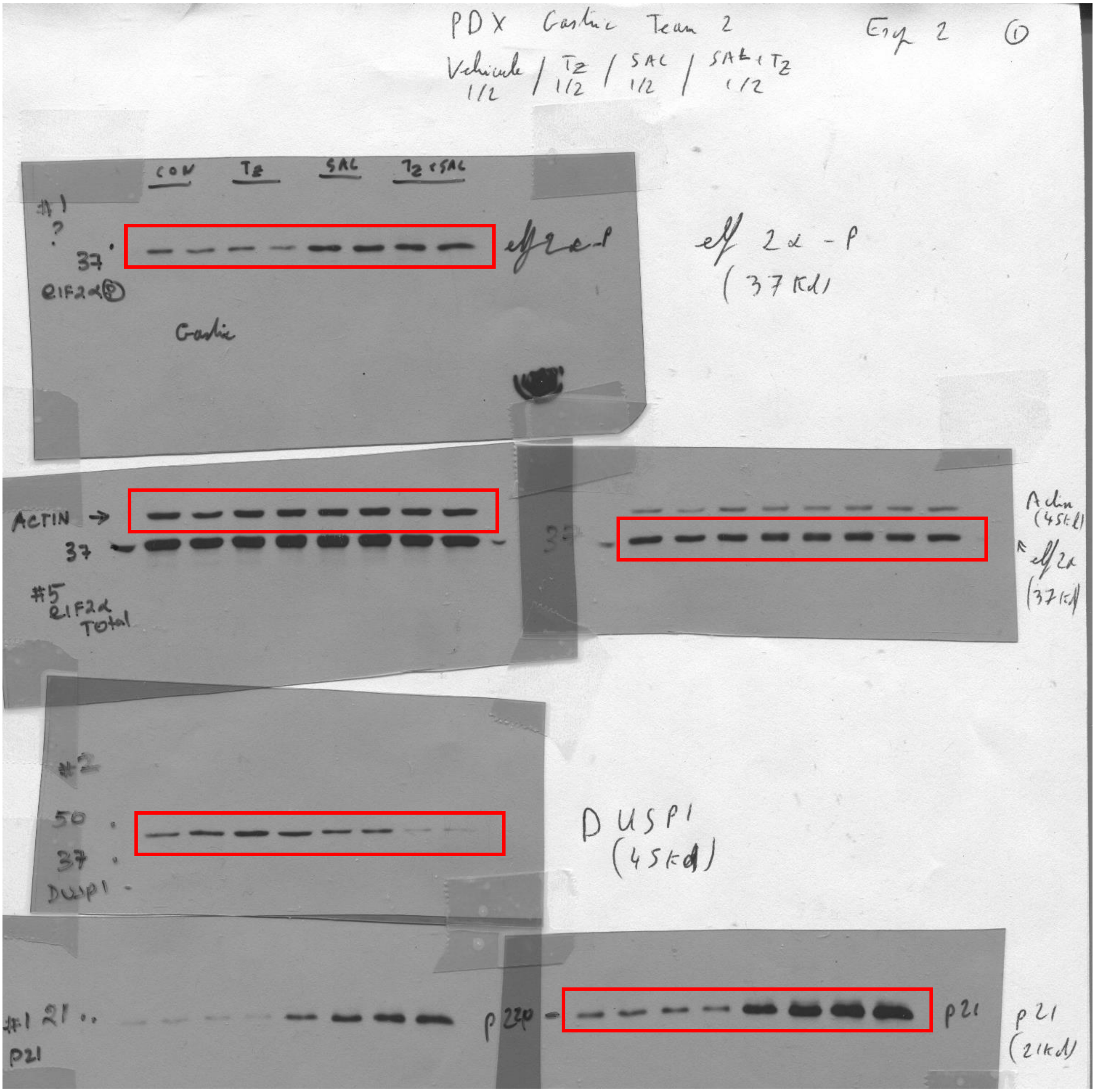

Blot of Supplementary Figure 7 (cont'd)

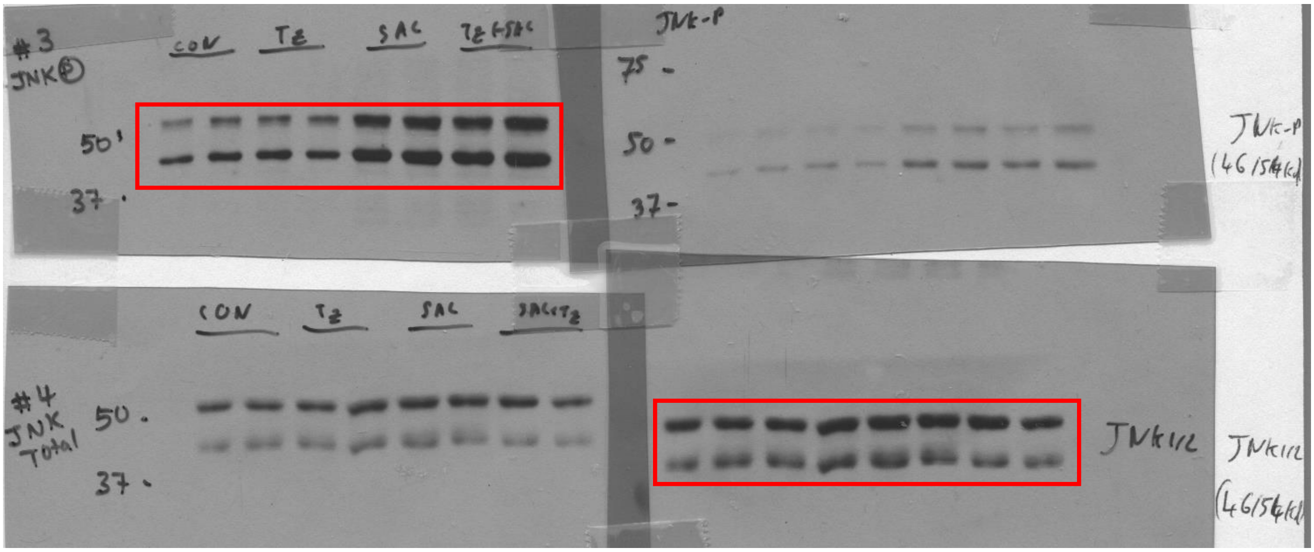

Blots of Supplementary Figure 7 (cont'd)

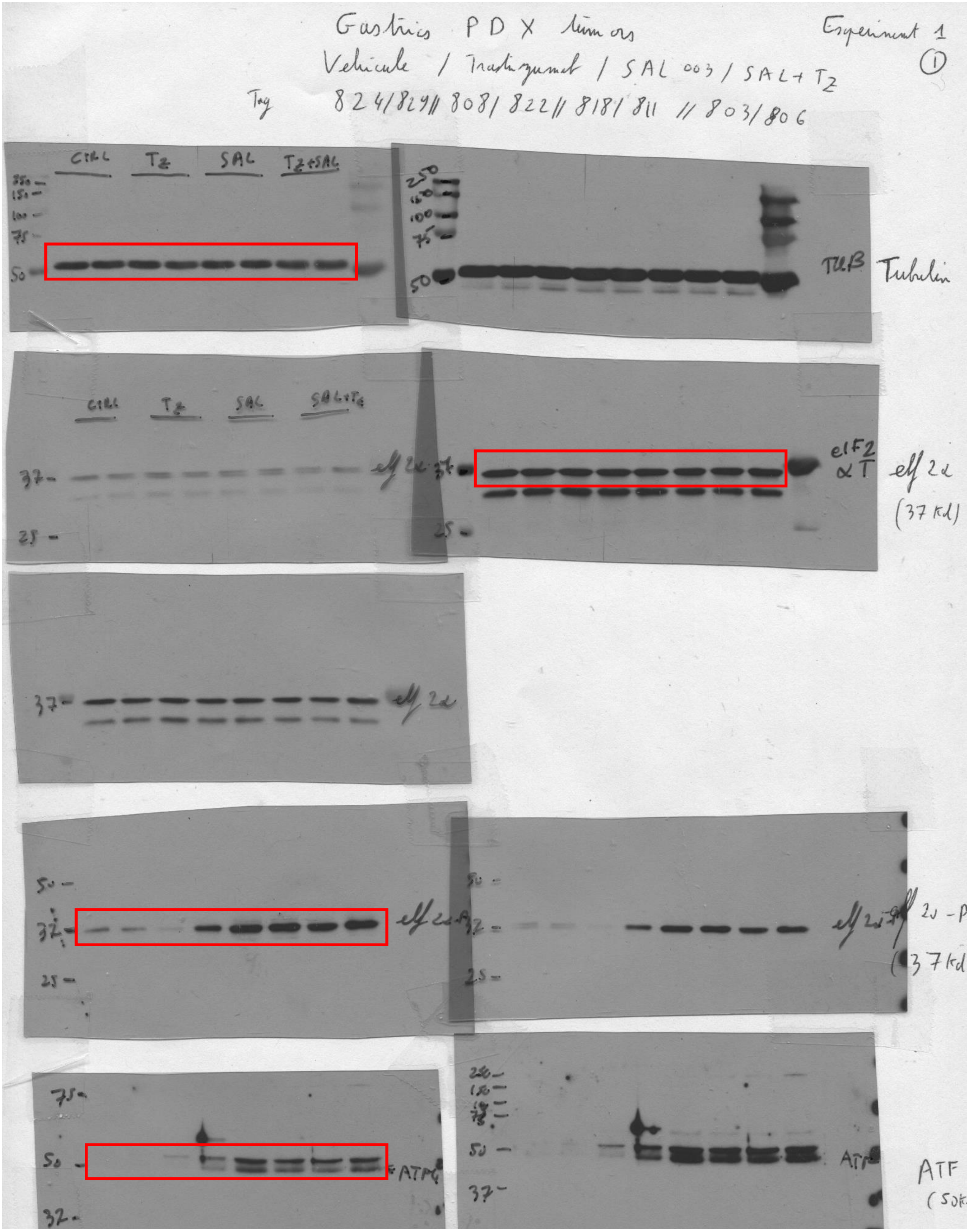

Blots of Supplementary Figure 7 (cont'd)

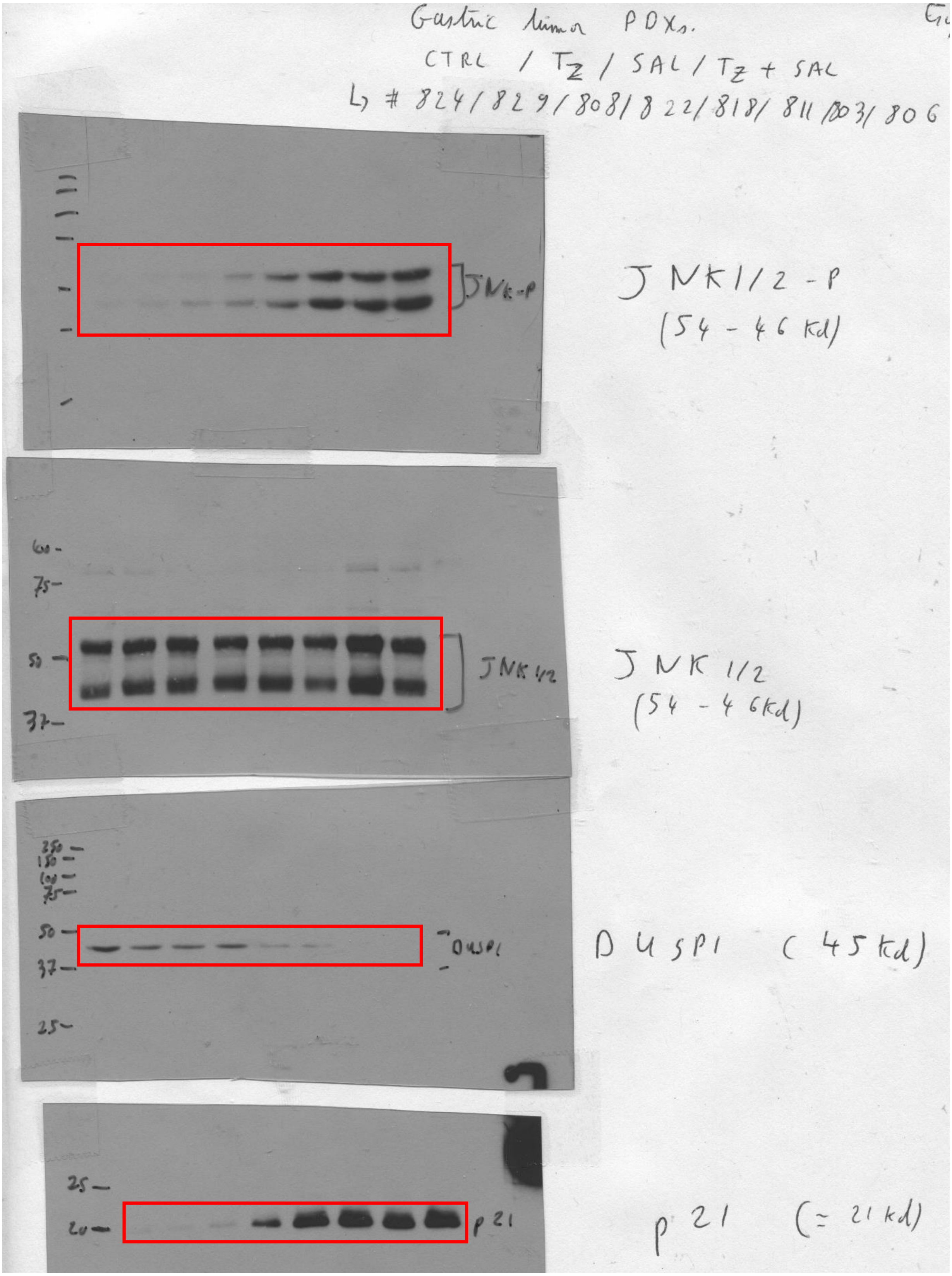

Supplement: Supplementary file 1 — Supplementary Information [file 41467_2019_10138_MOESM1_ESM.pdf]
